# Trends in the Concentration and Distribution of Health Care Expenditures in the US, 2001-2018

**DOI:** 10.1001/jamanetworkopen.2021.25179

**Published:** 2021-09-14

**Authors:** Maximilian Holle, Tory Wolff, Marc Herant

**Affiliations:** 1 Department of Health Policy, London School of Economics and Political Science, London, United Kingdom; 2 Recon Strategy, Redmond, Washington; 3 Recon Strategy, Cambridge, Massachusetts

## Abstract

**Importance:**

The concentration of health care expenditures has important implications for managing risk pools, drug benefit design, and care management.

**Objective:**

To examine trends in the concentration of health care spending in different population groups and expenditure categories in the US between 2001 and 2018.

**Design, Setting, and Participants:**

This study is a cross-sectional analysis of Medical Expenditure Panel Surveys (MEPS) collected between 2001 and 2018. The MEPS is a household survey of medical expenditures weighted to represent national estimates in the US. Respondents were a nationally representative sample of the US civilian noninstitutionalized population. Data analysis was performed from December 2020 to February 2021.

**Main Outcomes and Measures:**

The main outcome is the concentration of health care expenditures as measured by the cumulative percentage of health expenditure vs percentage of ranked population. This study reports trends in the distribution of populations across 4 concentration curve parameters: top 50% expenditure (high spenders), next 49% expenditure (medium spenders), next 1% expenditure (low spenders), and nonspenders.

**Results:**

The mean sample size of the MEPS surveys used in the analysis was 34 539 individuals, and the sample size varied between 30 461 and 39 165 individuals over the years studied. On the basis of data from 30 461 MEPS respondents (15 867 women [52.1%]; mean [SD] age, 38.9 [24.0] years) in 2018, the top 4.6% (95% CI, 4.3%-4.9%) of the US population by spending accounted for 50% of health care expenditures. Although this fraction varied across population groups or expenditure categories, it remained remarkably stable over time with one exception: the concentration of spending on prescription drugs. In 2001, one-half of all expenditures on prescription drugs were concentrated in 6.0% (95% CI, 5.6%-6.4%) of the US population, but by 2018, this proportion had decreased to 2.3% (95% CI, 2.1%-2.5%). This change does not appear to be associated with a change in the overall share of prescription drug expenses, which increased by only a small amount, from 20.4% in 2001 to 24.8% in 2018.

**Conclusions and Relevance:**

The overall concentration of health care expenditures remained stable between 2001 and 2018, but these findings suggest that there has been a sharp increase in the concentration of spending on prescription drugs in the US. This coincides with the genericization of many primary care drugs, along with a shift in focus of the biopharmaceutical industry toward high-cost specialty drugs targeted at smaller populations. If this trend continues, it will have implications for the minimum scale of risk-bearing and drug management needed to operate efficiently, as well as the optimal cost-sharing features of insurance products.

## Key Points

**Question:**

How has the concentration of health care spending in the US changed by population subgroup and expenditure type between 2001 and 2018?

**Findings:**

In this cross-sectional study, the overall concentration of health care expenditures remained stable, although there was a sharp increase in the concentration of spending on prescription drugs. In 2001, one-half of all expenditures on prescription drugs were concentrated in 6.0% of the US population, but by 2018, this proportion had decreased to 2.3% of the population.

**Meaning:**

These findings suggest that if this trend continues, it will have implications for the minimum scale of risk-bearing and drug management needed for health insurance plans to operate efficiently, as well as the optimal cost-sharing features of insurance products.

## Teaser

This cross-sectional study examines trends in the concentration of health care spending in different population groups and expenditure categories in the US between 2001 and 2018.

## Introduction

The concentration curve of cumulative percentage of health care expenditure vs percentage of ranked population is a powerful instrument in the analysis of health care costs. One key advantage is that it is intrinsically dimensionless and allows direct comparisons between populations of various types, in different expenditure categories, and across time periods irrespective of factors such as inflation or size adjustments. Despite that versatility, published analyses^1,2^ tend to focus on one or another feature of interest of the concentration curve rather than taking a broader view.

The yearly Medical Expenditure Panel Survey (MEPS) provides a large data set on health care spending in the US, with considerable granularity on expenditure categories and population groups. We used those data to construct a comprehensive set of concentration curves. We have considered not only the high-spender bracket but also analyzed other brackets, such as low spenders and nonspenders, as was done by Berk and Fang.^3^

In this study, we focus on a presentation of our most important findings; in general, for a given population segment or spending category, concentration curves have been remarkably stable over time, with one notable exception, prescription drug expenditures. However, we also provide a comprehensive set of summarized concentration curve parameters as a general resource to the community in eAppendix 1, eAppendix 2, and eAppendix 3 in the Supplement (a URL for a public repository containing these tables as Excel files is shown at the end of the article).

Our analysis sought to answer the following questions: How is the concentration of health care spending changing in the US over time? What are the differences in concentration curves between population groups (eg, income and insurance type)? What are the differences in concentration curves between expenditure categories (eg, outpatient vs inpatient)? How do these concentration curves change over time?

## Methods

The findings presented in this cross-sectional study come from an analysis of MEPS full-year consolidated data files, which contain data on surveys conducted between 1996 and 2018. MEPS is a survey of a nationally representative sample of the US civilian noninstitutionalized population that achieves a high level of accuracy in its expenditure estimates by validating household-reported expenditure data with data from health care facilities (eg, hospitals, outpatients facilities, and pharmacies). Cohen et al^4^ provide a detailed overview and discussion of MEPS.

MEPS data are publicly available, and there is no patient consent form available for download. Ethical review and informed consent were, therefore, not sought, in accordance with 45 CFR §46. This study follows the Strengthening the Reporting of Observational Studies in Epidemiology (STROBE) reporting guideline.

We used variables measuring health insurance status, income, and diagnostic group to study differences in expenditure concentrations across population subgroups. To classify individuals into insurance groups, we used the following hierarchy: (1) any public insurance at the end of the year, (2) any private insurance during the year, and (3) uninsured for the entire year. Public insurance holders were classified into mutually exclusive subgroups according to their insurance status at the end of the year (ie, Medicare Fee-for-Service, Medicare Advantage, Medicaid, and dual Medicare and Medicaid beneficiaries both <65 years and ≥65 years old). Individuals were assigned to income groups according to the total yearly income of their family. Income group classifications were based on family income relative to the poverty line and were the same as those used by previous analyses.^2^

Our calculation of total health care expenditures did not include vision aids and dental expenditures because they operate differently from core health care expenditure categories (ie, they are usually covered separately by insurers and have a high cash component, and a significant portion of spending has cosmetic intent). To study the concentration of different types of health care expenditures, we used MEPS health service category expenditure variables. Prescription drug expenditure includes all spending on prescribed medicines by the respondent and is derived from pharmacy claims data. Spending on drugs administered as part of a medical encounter is not included in prescription drug expenditures. Medicare spending data provide some insight into the relative magnitude of these categories; in 2018, $35.0 billion was spent on part B (physician and hospital administered) drugs, and $168.1 billion was spent on part D (prescription) drugs.^5^

Expenditure on emergency care resulting in an inpatient stay is included under inpatient care expenditure, and all other spending on emergency care is included under emergency care expenditure. Expenditures on home health and other medical supplies and equipment were combined under home health and equipment.

The expenditure brackets that we studied were mutually exclusive and were formed by ordering sampled persons by their expenditures and then allocating persons to brackets according to weighted cumulative expenditures. Note that in any category or subgroup analysis, persons were reordered and brackets were recalculated (ie, a specific person might be in a different bracket depending on the specific analysis). Before excluding dental and vision expenditures from the total expenditure calculation, we validated our methods by replicating the results of a recent report on health care expenditure concentration that also used MEPS data.^6^ We used a nonparametric bootstrap (with 1000 resamples) to obtain 95% CIs for the concentration parameters (Table).

**Table.** Decadal Change in Top 50% Expenditure Bracket (High Spenders) by US Population Subgroup and Expenditure Category From 2006-2008 to 2016-2018

Subgroup	Population, % (95% CI)		
	2006-2008	2016-2018	Ratio^a^
Age group, y			
0-64	3.9 (3.6-4.2)	3.5 (3.3-3.8)	0.92 (0.85-0.94)
≥65	10.7 (10.3-11.2)	9.4 (9.0-9.9)	0.88 (0.81-0.93)
Insurance status			
Private insurance	4.4 (4.1-4.7)	3.8 (3.5-4.1)	0.86 (0.78-0.98)
Medicare Fee-for-Service	10.0 (9.3-10.5)	9.4 (8.8-10.2)	0.93 (0.86-1.01)
Medicare Advantage	10.9 (10.2-11.7)	10.1 (9.3-10.7)	0.93 (0.85-1.02)
Medicaid	4.1 (3.7-4.4)	3.9 (3.6-4.2)	0.95 (0.86-1.05)
Dual Medicaid and Medicare			
Aged 0-64 y	10.5 (8.7-11.7)	12.0 (11.0-13.1)	1.14 (0.97-1.37)
Aged ≥65 y	12.3 (11.3-13.7)	11.2 (10.3-12.9)	0.91 (0.79-1.12)
Uninsured	2.8 (2.4-3.1)	1.6 (1.3-2.0)	0.57 (0.46-0.78)
Diabetes diagnosis	10.5 (9.8-11.4)	10.4 (9.8-11.1)	0.99 (0.90-1.08)
Annual household income times federal poverty line			
<1.00	3.6 (3.1-4.2)	4.4 (4.1-4.6)	1.20 (1.00-1.38)
1.01-1.24	4.5 (4.1-5.1)	4.7 (4.3-5.4)	1.04 (0.89-1.23)
1.25-1.99	4.2 (3.9-4.7)	4.1 (3.8-4.6)	0.98 (0.85-1.12)
2.0-3.99	4.6 (4.3-4.9)	3.9 (3.6-4.2)	0.86 (0.79-0.94)
≥4.00	5.3 (4.9-5.7)	4.9 (4.7-5.2)	0.93 (0.85-1.02)
Expenditure category			
Outpatient	4.6 (4.4-4.8)	4.6 (4.4-4.8)	0.99 (0.92-1.06)
Inpatient	1.0 (0.9-1)	0.9 (0.8-0.9)	0.87 (0.79-0.98)
Emergency department	1.3 (1.2-1.4)	1.6 (1.5-1.7)	1.21 (1.09-1.33)
Prescription drugs	5.1 (4.8-5.3)	2.4 (2.3-2.5)	0.47 (0.44-0.50)
Home health and equipment	0.3 (0.3-0.4)	0.5 (0.4-0.6)	1.54 (1.27-1.90)

^a^ Three-year averages (2006-2008 and 2016-2018) were used to calculate relative change.

Because of the effects related to a redesign of the NHIS sample following the 2000 decennial US Census, the main text presents only data starting from 2001; however, eAppendix 1, eAppendix 2, and eAppendix 3 in the Supplement contain MEPS data from before 2001. Data analysis was performed using RStudio statistical software version 1.3.1073 (RStudio Team) from December 2020 to February 2021.

## Results

The mean sample size of the MEPS surveys used in our analysis was 34 539 individuals, and the sample size varied between 30 461 and 39 165 individuals over the years studied. National estimates were based on survey weights and annual samples of MEPS survey respondents. In 2018, 52.1% of the survey population was female (15 867 women), and the mean (SD) age was 38.9 (24.0) years.

**Figure 1.**
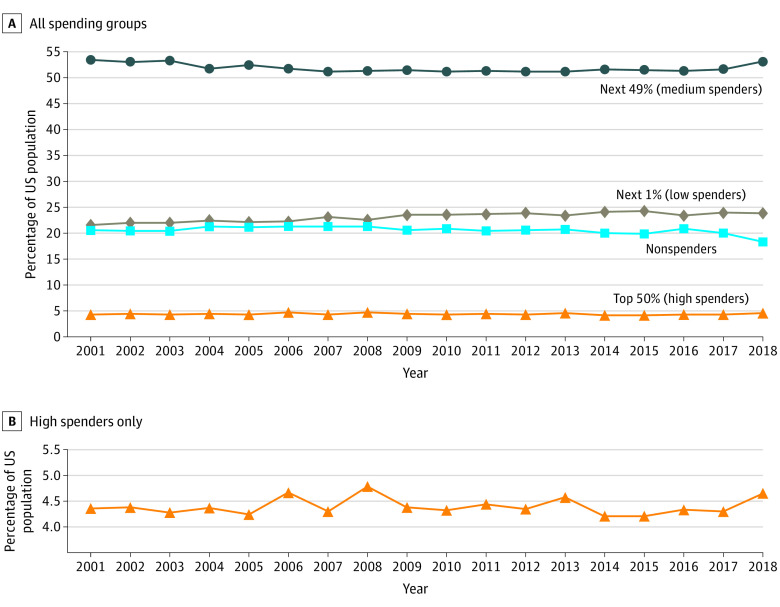
Distribution of US Population Across Cumulative Health Care Expenditure Brackets 2001-2018 Top 50% (high spenders) are those whose cumulative expenditures make up the top 50th percentile of expenditure. The next 49% (medium spenders) are those whose cumulative expenditures make up the 49th through first expenditure percentiles. The next 1% are those whose cumulative expenditures make up the first expenditure percentile. Nonspenders are those with no expenditures. Panel A shows data for all spending groups, and panel B shows data for the high spenders only.

Figure 1 shows the evolution over time of 4 expenditure brackets: the high spenders bracket, which accounts for the top 50% of total expenditures, the medium spenders bracket, which accounts for the next 49% of expenditures, the low spenders bracket, which accounts for the final 1% of expenditures, and the nonspenders bracket, which does not contribute at all to total expenditures. To give a sense of those brackets, in 2018, the mean (SD) spend in the top 50% bracket (accounting for 4.6% [95% CI, 4.3%-4.9%] of the US population) was $61 328 ($51 339), the transition point between the top 50% and next 49% brackets was $27 294, and the transition point between the next 49% and next 1% brackets was $529 (further details are available in eAppendix 1, eAppendix 2, and eAppendix 3 in the Supplement).

The concentration of health care expenditures across various expenditure brackets has been strikingly stable. For example, between 2001 and 2018, the top 50% bracket varied between 4.2% and 4.8% of the population, with small fluctuations consistent with statistical noise. This is a surprise given that, during this period, there have been substantial changes in US demographic characteristics (eg, an aging population and increasing diversity) and in health care coverage models (eg, the Patient Protection and Affordable Care Act). The proportion in the nonspender bracket is also consistent at approximately 20%, with a recent decrease in 2017 and 2018, which may or may not be confirmed in 2019. We broke down our analysis into 16 population subgroups and 5 expenditure categories looking at changes over a 10-year period focused on the top 50% bracket (Table; see eAppendix 1, eAppendix 2, and eAppendix 3 in the Supplement for additional brackets). What is notable is that although there are significant differences in the concentration of expenditures between groups and categories, the concentrations have been stable over time, with a few exceptions discussed later. That costs are much more concentrated among individuals with private insurance than for individuals with Medicare is not surprising and is consistent with prior findings.^2^

There are 3 exceptions to the stability of concentration. First, cost has become more concentrated among the uninsured. The Patient Protection and Affordable Care Act has been associated with more individuals with health issues getting insured, and the uninsured have become younger and healthier, which, in turn, leads to more concentrated costs.^7^ Second, the distribution of costs for home health and equipment has become less concentrated as the push to keep individuals out of the hospital with interventions at home has become stronger.

**Figure 2.**
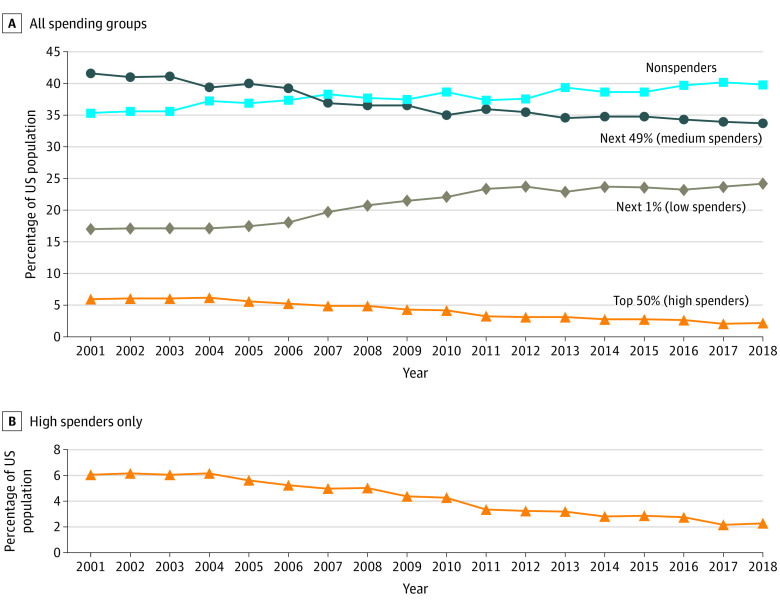
Distribution of US Population Across Cumulative Prescription Drugs Expenditure Brackets 2001-2018 Top 50% (high spenders) are those whose cumulative prescription drug expenditures make up the top 50th percentile of expenditure. The next 49% (medium spenders) are those whose cumulative prescription drug expenditures make up the 49th through first expenditure percentiles The next 1% (low spenders) are those whose cumulative prescription drug expenditures make up the first expenditure percentile. Nonspenders are those with no prescription drug expenditures. Panel A shows data for all spending groups, and panel B shows data for the high spenders only.

**Figure 3.**
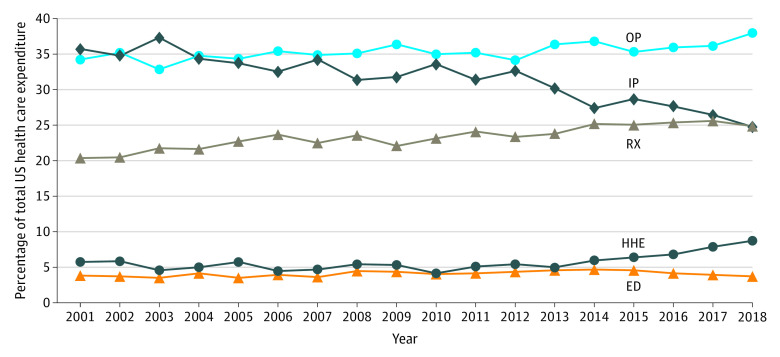
Health Service Category Shares of Total Health Care Expenditure 2001-2018 ED indicates emergency department; HHE, home health and equipment; IP, inpatient; OP, outpatient; RX, prescription drugs.

Third, for prescription drugs, there has been a sharp increase in the concentration of expenditures. Figure 2 shows that in 2001, 6.0% (95% CI, 5.6%-6.4%) of the population accounted for approximately one-half of all expenditures on prescription drugs. By 2018, this figure had decreased to 2.3% (95% CI, 2.1%-2.5%). This trend was observed across each of the population subgroups analyzed in the Table. Concomitantly, we found that the increasing concentration in prescription drug spending was associated with a large increase in the mean cost for high spenders. From 2001 to 2018, the mean (SD) expenditure for those in the top 50% of expenditure bracket increased from $5412 ($2646) to $30 523 ($28 583) (in 2018 US dollars). The change in concentration in prescription drugs expenditures does not appear to be associated with a change in the share of prescription drugs in overall health care costs, which, over the same time period has only increased by a small amount, from 20.4% in 2001 to 24.8% in 2018 (Figure 3).

## Discussion

As shown in this cross-sectional study, in any given year, slightly less than 5% of the US population accounts for 50% of health care expenditures. International comparisons with England^8^ or Germany^9^ suggest that this is a feature of health systems in high-income countries and that the US is not an outlier in this regard despite spending more per person on health care. At the same time, approximately 20% of the US population are nonspenders. What is remarkable is that despite more than 2 decades of explicit policy aimed at increasing preventative care with a goal of decreasing the incidence of acute, expensive conditions,^10,11^ these percentiles have hardly changed. This finding is consistent with what Berk and Fang^3^ have previously noted and, furthermore, carries over in nearly all population subgroup and expenditure category analyses.

One key exception to this constancy is the prescription drugs category, for which the expenditure concentration has increased greatly since around 2005 and continues through the final year of our analysis (2018). This is in contrast to an earlier study^12^ focused on a prior epoch (1996 to 2003) that linked growth in prescription drug spending with decreasing overall concentration in health care spending. Times have changed since. First, a rapid succession of patent expirations greatly decreased the price of a core set of primary care drugs in wide use, including statins, angiotensin receptor blockers, and selective serotonin reuptake inhibitors.^13^ Second, the biopharmaceutical industry pivoted strongly to high-priced drugs directed at specialty care for which patient populations are smaller.^14,15,16^ If anything, the present analysis underestimates this trend given that drugs administered as part of a medical encounter are not included under the prescription drugs category, a gap that will grow with the push toward ever more sophisticated therapeutics. There is a paucity of information on drug cost concentration in other countries that operate under more rigorously managed pricing regimes. One Canadian study^17^ has shown that in 2017, the top 50% expenditure bracket was made up of close to 5% of the population, compared with 2.2% in the US.

Despite the well-discussed high price of drugs in the US, drug expenditure as a proportion of total health care spending is not especially high in the US compared with other countries.^18^ However, from an actuarial point of view, the increased concentration of drug expenditures could become an issue. This is because in smaller risk pools (eg, medium-size, self-insured employers or smaller plans, of which there are many in the US), small sample size effects (eg, few individuals who need a gene therapy) can have a disproportionate effect on financial performance. There are ways to mitigate this effect through stop-loss reinsurance, but even then, the impact on premiums of having a stop-loss claim is likely to be long-lasting. In addition, employers that were previously large enough to avoid a stop-loss add-on may no longer be so. Finally, pharmacy benefit plans designed for more evenly distributed expenditures can have unintended effects on patients receiving high-cost therapies, such as very large financial burdens arising out of benefit cost-sharing features. Tackling the needs of these special case patients effectively and efficiently requires tremendous scale in population coverage. If the trend of increasing concentration of drug costs continues, it will become an additional driver of consolidation of the insurance market and will increase the costs of self-insurance for most except the biggest employers.

### Limitations

This study had several limitations. First, because MEPS is limited to the civilian, noninstitutionalized population, spending by institutionalized individuals (eg, individuals in nursing homes) is not included. This limits the generalizability of our findings to these demographic groups. MEPS utilization data are also based on household reports, which tend to underreport utilization. Some expenditure estimates are, therefore, slightly lower than those of comparable surveys.^4^ In addition, spending on drugs administered as part of a medical encounter was not captured in the data used. Our findings, therefore, provide somewhat limited insight into changes into the overall concentration of spending on pharmaceutical products. MEPS data were also available only up to 2018; as a result, our findings are not fully current.

## Conclusions

This study described a variety of trends in the concentration and distribution of health care expenditures in the US between 2001 and 2018. During this period, the overall concentration of health care expenditures remained stable, but there was a sharp increase in the concentration of spending on prescription drugs. In 2001, one-half of all expenditures on prescription drugs were concentrated in 6.0% of the US population, but by 2018, this proportion had decreased to 2.3%. If this trend continues, it will have implications for the minimum scale of risk-bearing and drug management needed for health insurance plans to operate efficiently. It will also place constraints on optimal cost-sharing features of insurance products.

## Notes

Supplement. **eAppendix 1.** Concentration Parameters by Expenditure Category and Population Subgroup **eAppendix 2.** Prescription Drug Expenditure Concentration Parameters for Population Subgroups **eAppendix 3.** Expenditure Category Shares of Total Expenditure 1996-2018
