# Supplementary material for: Trends in the Concentration and Distribution of Health Care Expenditures in the US, 2001-2018
Source: JAMA Netw Open. 2021 Sep 14;4(9):e2125179. doi: 10.1001/jamanetworkopen.2021.25179 (PMC8441588; doi:10.1001/jamanetworkopen.2021.25179)
Supplement: Supplement. — eAppendix 1. Concentration Parameters by Expenditure Category and Population Subgroup eAppendix 2. Prescription Drug Expenditure Concentration Parameters for Population Subgroups eAppendix 3. Expenditure Category Shares of Total Expenditure 1996-2018 [file jamanetwopen-e2125179-s001.pdf]

Supplemental Online Content

Holle M, Wolff T, Herant M. Trends in the concentration and distribution of health care expenditures in the US, 2001-2018. *JAMA Netw Open*. 2021;4(9):e2125179. doi:10.1001/jamanetworkopen.2021.25179

eAppendix 1. Concentration Parameters by Expenditure Category and Population Subgroup

eAppendix 2. Prescription Drug Expenditure Concentration Parameters for Population Subgroups

eAppendix 3. Expenditure Category Shares of Total Expenditure 1996-2018

This supplemental material has been provided by the authors to give readers additional information about their work.

© 2021 Holle M et al. *JAMA Network Open.*

eAppendix 1: Concentration Parameters by Expenditure Category and Population Subgroup
**Introduction**
These tables present data on concentration parameters for different population groups and expenditure categories between 1996-2018. These tables are also available as Microsoft Excel files from a public repository (https://github.com/recon-strategy/concentration-parameters).
The "Summaries" sheet provides an overview of the studied population subgroups and expenditure categories.
**Contents**
Variable descriptions
Summaries
2018 Comparison
US_pop
US_pop_0_65
US_pop_65+
OP_EXP
IP_EXP
ED_EXP
RX_EXP
HHE_EXP
Any_private
Any_medicare
FFS
MA
MCAID
Dual
Unins
Diabetics
IncGrp1
IncGrp2
IncGrp3
IncGrp4

© 2021 Holle M et al. *JAMA Network Open.*

IncGrp5	
**Variable Descriptions**	
**Variable**	**Description**
Top 50%	Percentage of the population whose cumulative expenditures make up the top 50th percentile of expenditure
Next 49%	Percentage of the population whose cumulative expenditures make up the 49th-1st expenditure percentiles
Next 1%	Percentage of the population whose cumulative expenditures make up the 1st expenditure percentile
Non-spenders	Percentage of the population with no expenditures
Cutoff_50[a]	Lowest individual expenditure in the top 50% of cumulative expenditure
Cutoff_49[a]	Lowest individual expenditure in the top 99% of cumulative expenditure
Mean_50[a]	Mean expenditure of the top 50% cumulative expenditure bracket
%_total	Expenditure category share of total expenditure (where applicable)
[a]*not adjusted for inflation*	

© 2021 Holle M et al. *JAMA Network Open.*

**Summaries**		
**Subgroup/expenditure category**	**Population**	**Expenditure variable**
US_pop	Full US population	Total expenditure - vision aids - dental
US_Pop (0-64)	US Population (0-64)	Total expenditure - vision aids - dental
US_Pop (65+)	US Population (65+)	Total expenditure - vision aids - dental
OP_EXP	Full US population	Office based visits + expenditure on outpatient visits
IP_EXP	Full US population	Inpatient
ED_EXP	Full US population	Emergency room
RX_EXP	Full US population	Prescription medicines
HHE_EXP	Full US population	Home health expenditures + other medical supplies and equipment expenditures

© 2021 Holle M et al. *JAMA Network Open.*

any_private	Individual had any private insurance coverage at any time during the year and was not covered by public insurance	Total expenditure - vision aids - dental
any_medicare	Covered by Medicare	Total expenditure - vision aids - dental
FFS	Covered by Medicare but not Medicare Managed Care or Medicaid	Total expenditure - vision aids - dental
MA	Covered by Medicare Managed Care/Medicare Advantage	Total expenditure - vision aids - dental
MCAID	Covered by Medicaid or SCHIP	Total expenditure - vision aids - dental
Dual	Covered by both Medicare and Medicaid at time of interview	Total expenditure - vision aids - dental
Unins	Individual was uninsured during all of 2018	Total expenditure - vision aids - dental
Diabetics	Diabetes diagnosis at time of interview (pre 2018 only over 18s asked)	Total expenditure - vision aids - dental
IncGrp1	Family income less than 100% of poverty line	Total expenditure - vision aids - dental
IncGrp2	Family income between 100% - 125% of poverty line	Total expenditure - vision aids - dental

© 2021 Holle M et al. *JAMA Network Open.*

IncGrp3	Family income between 125% - 199% of poverty line	Total expenditure - vision aids - dental
IncGrp4	Family income between 200% - 399% of poverty line	Total expenditure - vision aids - dental
IncGrp5	Family income greater than 400% of poverty line	Total expenditure - vision aids - dental
Note: for some subgroups, data starts after 1996 due to methodological changes/data availability.		

© 2021 Holle M et al. *JAMA Network Open.*

**2018 Comparison**									
**Segment**	**Year**	**Top 50%**	**Next 49%**	**Next 1%**	**Non-spenders**	**Cutoff_50**	**Cutoff_49**	**Mean_50**	**%_total**
US_pop	2018	4.6%	53.2%	23.9%	18.3%	$ 27,294	$ 529	$ 61,328	100.0%
US_Pop (0-64)	2018	3.7%	52.1%	23.0%	21.2%	$ 25,538	$ 404	$ 60,536	100.0%
US_Pop (65+)	2018	9.5%	68.7%	17.7%	4.0%	$ 30,263	$ 1,325	$ 63,084	100.0%
OP_EXP	2018	4.8%	55.6%	16.4%	23.2%	$ 9,437	$ 233	$ 23,046	37.9%
IP_EXP	2018	0.8%	5.0%	1.0%	93.1%	$ 38,442	$ 2,939	$ 82,810	24.7%
ED_EXP	2018	1.6%	10.0%	2.0%	86.3%	$ 3,296	$ 201	$ 6,546	3.7%
RX_EXP	2018	2.3%	33.8%	24.2%	39.8%	$ 13,182	$ 175	$ 30,523	24.8%
HH_EXP	2018	0.7%	9.5%	5.7%	84.1%	$ 17,281	$ 332	$ 36,549	8.8%
any_private	2018	4.6%	56.1%	23.3%	16.0%	$ 26,191	$ 513	$ 60,207	100.0%
any_medicare	2018	9.9%	68.7%	17.8%	3.6%	$ 32,144	$ 1,419	$ 64,487	100.0%
FFS	2018	10.1%	69.5%	17.1%	3.4%	$ 29,323	$ 1,357	$ 58,442	100.0%
MA	2018	9.9%	70.2%	17.4%	2.5%	$ 29,369	$ 1,319	$ 61,339	100.0%
MCAID	2018	4.1%	48.8%	28.2%	18.8%	$ 34,109	$ 483	$ 74,227	100.0%
Dual	2018	11.8%	63.5%	20.2%	4.5%	$ 47,696	$ 2,347	$ 85,752	100.0%
Unins	2018	1.6%	32.5%	12.6%	53.3%	$ 12,845	$ 160	$ 32,170	100.0%
Diabetics	2018	10.6%	67.9%	18.6%	2.9%	$ 38,243	$ 1,927	$ 78,351	100.0%
IncGrp1	2018	4.4%	45.7%	26.9%	23.0%	$ 32,753	$ 569	$ 70,785	100.0%
IncGrp2	2018	4.7%	49.2%	24.4%	21.7%	$ 33,580	$ 526	$ 63,793	100.0%
IncGrp3	2018	4.4%	48.6%	25.8%	21.2%	$ 29,834	$ 491	$ 65,299	100.0%
IncGrp4	2018	4.2%	51.7%	24.0%	20.2%	$ 27,539	$ 491	$ 62,056	100.0%
IncGrp5	2018	5.2%	58.1%	22.2%	14.5%	$ 24,355	$ 550	$ 55,875	100.0%

© 2021 Holle M et al. *JAMA Network Open.*

US_pop							
**Year**	**Top 50%**	**Next 49%**	**Next 1%**	**Non-spenders**	**Cutoff_50**	**Cutoff_49**	**Mean_50**
2018	4.6%	53.2%	23.9%	18.3%	$ 27,294	$ 529	$ 61,328
2017	4.3%	51.7%	23.9%	20.0%	$ 25,802	$ 469	$ 57,607
2016	4.3%	51.4%	23.5%	20.8%	$ 23,919	$ 445	$ 53,751
2015	4.2%	51.6%	24.3%	19.9%	$ 24,620	$ 432	$ 54,590
2014	4.2%	51.6%	24.2%	20.0%	$ 22,094	$ 419	$ 51,446
2013	4.6%	51.3%	23.5%	20.7%	$ 20,499	$ 400	$ 45,776
2012	4.3%	51.2%	23.9%	20.5%	$ 20,306	$ 389	$ 47,235
2011	4.4%	51.3%	23.8%	20.5%	$ 20,140	$ 383	$ 45,781
2010	4.3%	51.2%	23.6%	20.9%	$ 19,831	$ 368	$ 44,879
2009	4.4%	51.5%	23.5%	20.6%	$ 18,599	$ 376	$ 43,092
2008	4.8%	51.3%	22.6%	21.3%	$ 16,282	$ 359	$ 37,136
2007	4.3%	51.3%	23.2%	21.2%	$ 17,268	$ 339	$ 39,836
2006	4.7%	51.8%	22.3%	21.2%	$ 14,811	$ 323	$ 34,453
2005	4.2%	52.5%	22.1%	21.1%	$ 15,441	$ 321	$ 36,784
2004	4.4%	51.8%	22.5%	21.4%	$ 14,272	$ 303	$ 33,121
2003	4.3%	53.4%	22.0%	20.4%	$ 13,287	$ 282	$ 31,435
2002	4.4%	53.1%	22.0%	20.5%	$ 12,293	$ 258	$ 29,813
2001	4.4%	53.5%	21.6%	20.6%	$ 11,163	$ 234	$ 26,574
2000	3.7%	51.8%	21.4%	23.1%	$ 11,033	$ 207	$ 26,117
1999	3.4%	52.2%	21.9%	22.5%	$ 10,754	$ 192	$ 27,573
1998	3.4%	51.6%	22.2%	22.8%	$ 10,653	$ 184	$ 26,960
1997	3.2%	51.7%	22.9%	22.2%	$ 11,967	$ 173	$ 28,068
1996	3.0%	52.7%	23.3%	21.0%	$ 12,417	$ 172	$ 30,348

© 2021 Holle M et al. *JAMA Network Open.*

US_pop (0-64)							
Year	Top 50%	Next 49%	Next 1%	Non-spenders	Cutoff_50	Cutoff_49	Mean_50
2018	3.7%	52.1%	23.0%	21.2%	$ 25,538	$ 404	$ 60,536
2017	3.4%	50.8%	22.7%	23.1%	$ 23,196	$ 356	$ 54,242
2016	3.5%	50.5%	22.2%	23.9%	$ 20,685	$ 339	$ 50,587
2015	3.3%	50.8%	23.2%	22.7%	$ 22,328	$ 334	$ 52,197
2014	3.2%	50.9%	23.1%	22.7%	$ 19,790	$ 324	$ 49,265
2013	3.6%	50.5%	22.5%	23.4%	$ 18,883	$ 307	$ 44,184
2012	3.5%	50.2%	23.0%	23.3%	$ 18,514	$ 303	$ 46,249
2011	3.7%	50.4%	22.9%	23.1%	$ 18,235	$ 300	$ 44,818
2010	3.7%	50.7%	22.3%	23.4%	$ 16,863	$ 291	$ 40,340
2009	3.7%	50.8%	22.4%	23.1%	$ 16,312	$ 294	$ 40,064
2008	4.0%	50.5%	21.7%	23.8%	$ 14,377	$ 283	$ 34,594
2007	3.5%	50.7%	22.1%	23.6%	$ 15,478	$ 271	$ 37,733
2006	4.0%	51.1%	21.2%	23.7%	$ 12,880	$ 252	$ 31,216
2005	3.7%	51.8%	21.0%	23.5%	$ 13,384	$ 254	$ 33,776
2004	3.8%	51.2%	21.2%	23.9%	$ 11,806	$ 235	$ 28,799
2003	3.8%	52.9%	20.6%	22.7%	$ 10,644	$ 221	$ 26,760
2002	3.9%	52.8%	20.7%	22.7%	$ 10,120	$ 202	$ 26,197
2001	3.9%	53.1%	20.1%	22.8%	$ 9,016	$ 185	$ 22,373
2000	3.3%	51.1%	20.1%	25.4%	$ 9,446	$ 165	$ 23,113
1999	3.1%	51.7%	20.4%	24.7%	$ 8,721	$ 154	$ 23,586
1998	3.1%	51.1%	20.6%	25.1%	$ 8,584	$ 145	$ 22,712
1997	2.9%	51.6%	21.2%	24.4%	$ 9,875	$ 139	$ 24,695
1996	2.5%	52.7%	21.9%	22.9%	$ 10,595	$ 138	$ 28,523

© 2021 Holle M et al. *JAMA Network Open.*

US_pop (65+)							
Year	Top 50%	Next 49%	Next 1%	Non-spenders	Cutoff_50	Cutoff_49	Mean_50
2018	9.5%	68.7%	17.7%	4.0%	$ 30,263	$ 1,325	$ 63,084
2017	9.3%	67.9%	18.6%	4.2%	$ 29,795	$ 1,215	$ 62,598
2016	9.4%	67.8%	18.4%	4.5%	$ 30,554	$ 1,247	$ 57,473
2015	9.5%	68.1%	18.2%	4.2%	$ 30,074	$ 1,176	$ 57,693
2014	10.2%	67.5%	18.2%	4.1%	$ 25,180	$ 1,184	$ 51,230
2013	10.2%	67.7%	17.3%	4.8%	$ 23,607	$ 1,131	$ 47,204
2012	9.5%	68.2%	18.4%	3.9%	$ 24,403	$ 992	$ 48,683
2011	9.4%	69.0%	17.8%	3.8%	$ 24,376	$ 1,025	$ 47,525
2010	9.4%	68.5%	17.9%	4.1%	$ 26,500	$ 1,168	$ 52,608
2009	9.6%	68.5%	17.9%	3.9%	$ 23,988	$ 1,137	$ 48,659
2008	11.1%	69.9%	15.4%	3.6%	$ 21,127	$ 1,082	$ 41,526
2007	10.4%	69.5%	16.1%	4.0%	$ 21,941	$ 1,142	$ 43,775
2006	10.5%	71.0%	14.9%	3.6%	$ 19,644	$ 1,057	$ 39,412
2005	9.5%	70.8%	15.8%	3.9%	$ 21,145	$ 1,010	$ 40,813
2004	10.3%	70.8%	15.7%	3.2%	$ 19,539	$ 1,025	$ 39,062
2003	9.7%	70.3%	16.3%	3.8%	$ 19,662	$ 901	$ 38,356
2002	9.9%	70.5%	15.8%	3.8%	$ 17,342	$ 901	$ 35,757
2001	9.4%	68.8%	17.4%	4.5%	$ 16,563	$ 742	$ 33,017
2000	8.2%	68.3%	17.9%	5.6%	$ 17,319	$ 633	$ 32,688
1999	7.1%	68.4%	19.0%	5.4%	$ 17,724	$ 600	$ 36,046
1998	7.6%	68.5%	18.7%	5.3%	$ 16,444	$ 564	$ 34,566
1997	6.9%	66.8%	20.2%	6.2%	$ 17,231	$ 529	$ 34,633
1996	7.3%	66.8%	19.2%	6.6%	$ 16,192	$ 525	$ 32,895

© 2021 Holle M et al. *JAMA Network Open.*

OP								
Year	Top 50%	Next 49%	Next 1%	Non-spenders	Cutoff_50	Cutoff_49	Mean_50	%_total
2018	4.8%	55.6%	16.4%	23.2%	$ 9,437	$ 233	$ 23,046	37.9%
2017	4.7%	54.4%	15.7%	25.3%	$ 8,057	$ 203	$ 19,963	36.1%
2016	4.3%	53.6%	15.7%	26.4%	$ 7,844	$ 189	$ 18,891	35.9%
2015	4.7%	53.7%	16.1%	25.5%	$ 7,652	$ 181	$ 18,158	35.3%
2014	4.4%	53.9%	16.1%	25.6%	$ 7,618	$ 180	$ 18,674	36.8%
2013	4.6%	53.5%	15.5%	26.4%	$ 7,050	$ 171	$ 16,124	36.4%
2012	4.6%	53.1%	15.6%	26.8%	$ 6,309	$ 154	$ 14,851	34.1%
2011	4.7%	52.9%	15.6%	26.8%	$ 6,823	$ 156	$ 15,335	35.2%
2010	4.5%	52.7%	15.8%	27.1%	$ 6,421	$ 152	$ 15,043	34.9%
2009	4.4%	52.4%	16.2%	27.0%	$ 6,652	$ 152	$ 15,801	36.4%
2008	4.9%	52.4%	15.4%	27.3%	$ 5,556	$ 141	$ 12,607	35.1%
2007	4.3%	52.0%	15.5%	28.1%	$ 5,902	$ 135	$ 14,006	34.8%
2006	4.8%	51.8%	15.9%	27.5%	$ 5,165	$ 125	$ 12,074	35.4%
2005	4.6%	52.3%	15.5%	27.7%	$ 5,215	$ 124	$ 12,109	34.3%
2004	4.7%	52.0%	15.4%	27.9%	$ 5,122	$ 118	$ 11,267	34.7%
2003	5.2%	52.9%	15.0%	26.9%	$ 4,195	$ 110	$ 9,012	32.8%
2002	5.1%	52.7%	15.3%	26.9%	$ 4,060	$ 104	$ 9,262	35.1%
2001	5.0%	52.8%	14.3%	27.9%	$ 3,640	$ 95	$ 8,070	34.2%
2000	4.7%	51.4%	13.5%	30.4%	$ 3,170	$ 82	$ 7,098	32.1%
1999	5.0%	51.8%	13.8%	29.4%	$ 2,880	$ 75	$ 6,260	31.5%
1998	4.6%	51.3%	14.2%	29.9%	$ 3,220	$ 76	$ 6,888	34.0%
1997	4.4%	52.2%	13.9%	29.4%	$ 3,000	$ 72	$ 6,566	31.8%
1996	4.7%	52.4%	14.4%	28.5%	$ 2,940	$ 72	$ 6,224	32.4%

© 2021 Holle M et al. *JAMA Network Open.*

IP								
**Year**	**Top 50%**	**Next 49%**	**Next 1%**	**Non-spenders**	**Cutoff_50**	**Cutoff_49**	**Mean_50**	**%_total**
2018	0.8%	5.0%	1.0%	93.1%	$ 38,442	$ 2,939	$ 82,810	24.7%
2017	0.8%	4.8%	0.9%	93.5%	$ 37,599	$ 3,143	$ 77,761	26.4%
2016	0.9%	5.0%	0.8%	93.3%	$ 33,062	$ 3,200	$ 69,315	27.7%
2015	0.8%	4.7%	0.9%	93.6%	$ 39,623	$ 3,378	$ 78,759	28.6%
2014	0.8%	4.7%	1.0%	93.4%	$ 35,863	$ 2,887	$ 71,753	27.4%
2013	1.0%	5.4%	1.0%	92.7%	$ 29,830	$ 2,720	$ 63,350	30.2%
2012	1.0%	5.4%	0.9%	92.8%	$ 33,087	$ 3,205	$ 69,070	32.6%
2011	0.9%	5.2%	0.9%	93.0%	$ 33,425	$ 3,084	$ 65,273	31.4%
2010	1.0%	5.2%	0.9%	92.9%	$ 36,575	$ 2,838	$ 64,119	33.5%
2009	0.9%	5.2%	0.9%	93.0%	$ 34,195	$ 2,914	$ 63,287	31.7%
2008	1.0%	5.3%	0.9%	92.8%	$ 28,931	$ 2,671	$ 60,122	31.4%
2007	1.0%	5.6%	0.9%	92.6%	$ 30,365	$ 2,914	$ 59,311	34.2%
2006	1.0%	5.3%	0.9%	92.8%	$ 27,837	$ 2,686	$ 54,249	32.5%
2005	0.9%	5.6%	0.9%	92.5%	$ 28,108	$ 2,203	$ 58,354	33.7%
2004	0.9%	5.8%	0.9%	92.5%	$ 28,021	$ 2,353	$ 55,204	34.4%
2003	0.8%	5.7%	0.9%	92.6%	$ 29,067	$ 2,296	$ 61,772	37.3%
2002	0.9%	5.7%	0.9%	92.5%	$ 24,405	$ 2,052	$ 50,686	34.8%
2001	1.0%	5.8%	0.9%	92.3%	$ 19,807	$ 1,727	$ 41,195	35.7%
2000	0.9%	5.7%	1.0%	92.4%	$ 22,586	$ 1,811	$ 45,818	40.9%
1999	0.8%	5.5%	0.9%	92.8%	$ 23,127	$ 1,750	$ 46,047	40.2%
1998	0.8%	5.5%	1.0%	92.8%	$ 24,040	$ 1,629	$ 46,938	41.8%
1997	0.8%	5.6%	1.0%	92.5%	$ 22,292	$ 1,436	$ 43,907	42.6%
1996	0.7%	5.5%	0.9%	92.9%	$ 23,686	$ 1,670	$ 54,032	42.8%

© 2021 Holle M et al. *JAMA Network Open.*

ED								
Year	Top 50%	Next 49%	Next 1%	Non-spenders	Cutoff_50	Cutoff_49	Mean_50	%_total
2018	1.6%	10.0%	2.0%	86.3%	$ 3,296	$ 201	$ 6,546	3.7%
2017	1.6%	9.8%	1.9%	86.7%	$ 3,055	$ 184	$ 6,060	4.0%
2016	1.5%	9.6%	2.0%	86.9%	$ 3,138	$ 183	$ 6,690	4.2%
2015	1.3%	10.3%	2.1%	86.4%	$ 3,475	$ 190	$ 7,866	4.6%
2014	1.5%	9.9%	2.0%	86.6%	$ 3,075	$ 185	$ 6,706	4.7%
2013	1.5%	10.0%	1.9%	86.6%	$ 2,899	$ 177	$ 6,643	4.6%
2012	1.6%	9.3%	1.9%	87.2%	$ 2,981	$ 170	$ 5,490	4.5%
2011	1.4%	9.2%	1.8%	87.6%	$ 2,731	$ 175	$ 5,874	4.2%
2010	1.1%	8.7%	1.7%	88.4%	$ 3,270	$ 175	$ 7,214	4.1%
2009	1.4%	9.4%	1.8%	87.3%	$ 2,953	$ 157	$ 6,481	4.4%
2008	1.1%	9.2%	1.9%	87.7%	$ 3,098	$ 152	$ 6,563	4.5%
2007	1.4%	9.0%	1.8%	87.8%	$ 2,319	$ 128	$ 4,788	3.7%
2006	1.4%	9.5%	1.7%	87.4%	$ 2,210	$ 135	$ 4,560	4.0%
2005	1.5%	9.7%	1.7%	87.1%	$ 1,797	$ 126	$ 3,719	3.5%
2004	1.3%	10.0%	1.9%	86.8%	$ 2,123	$ 120	$ 4,530	4.2%
2003	1.7%	10.0%	1.7%	86.7%	$ 1,501	$ 115	$ 3,037	3.6%
2002	1.5%	10.2%	1.7%	86.6%	$ 1,404	$ 98	$ 3,313	3.8%
2001	1.5%	10.1%	1.6%	86.8%	$ 1,296	$ 96	$ 2,962	3.9%
2000	1.4%	8.8%	1.5%	88.4%	$ 1,209	$ 82	$ 2,501	3.4%
1999	1.4%	8.5%	1.3%	88.7%	$ 1,014	$ 80	$ 2,142	3.2%
1998	1.6%	8.4%	1.3%	88.8%	$ 977	$ 77	$ 1,945	3.3%
1997	1.3%	8.8%	1.4%	88.4%	$ 1,122	$ 81	$ 2,789	3.6%
1996	1.3%	8.9%	1.3%	88.4%	$ 1,038	$ 78	$ 2,290	3.4%

© 2021 Holle M et al. *JAMA Network Open.*

RX								
**Year**	**Top 50%**	**Next 49%**	**Next 1%**	**Non-spenders**	**Cutoff_50**	**Cutoff_49**	**Mean_50**	**%_total**
2018	2.3%	33.8%	24.2%	39.8%	$ 13,182	$ 175	$ 30,523	24.8%
2017	2.2%	33.9%	23.7%	40.2%	$ 11,973	$ 165	$ 29,136	25.6%
2016	2.7%	34.3%	23.2%	39.7%	$ 9,530	$ 163	$ 21,573	25.4%
2015	2.8%	34.8%	23.7%	38.7%	$ 8,745	$ 152	$ 19,628	25.1%
2014	2.8%	34.8%	23.8%	38.6%	$ 8,198	$ 140	$ 18,375	25.1%
2013	3.2%	34.6%	22.9%	39.3%	$ 6,914	$ 127	$ 15,395	23.8%
2012	3.2%	35.5%	23.7%	37.6%	$ 6,335	$ 122	$ 15,325	23.4%
2011	3.3%	35.9%	23.4%	37.3%	$ 6,151	$ 128	$ 15,988	24.1%
2010	4.2%	35.0%	22.0%	38.7%	$ 4,902	$ 125	$ 10,988	23.2%
2009	4.4%	36.6%	21.6%	37.5%	$ 4,771	$ 112	$ 9,852	22.1%
2008	5.0%	36.5%	20.8%	37.7%	$ 4,215	$ 118	$ 8,142	23.5%
2007	5.0%	37.0%	19.8%	38.3%	$ 3,943	$ 112	$ 7,785	22.5%
2006	5.2%	39.3%	18.1%	37.4%	$ 3,459	$ 105	$ 6,783	23.6%
2005	5.6%	40.0%	17.5%	36.9%	$ 3,155	$ 102	$ 6,184	22.7%
2004	6.2%	39.4%	17.1%	37.3%	$ 2,809	$ 90	$ 5,416	21.7%
2003	6.1%	41.1%	17.2%	35.6%	$ 2,740	$ 81	$ 5,164	21.7%
2002	6.2%	41.0%	17.2%	35.6%	$ 2,315	$ 75	$ 4,436	20.4%
2001	6.0%	41.6%	17.1%	35.3%	$ 2,115	$ 68	$ 3,897	20.4%
2000	5.7%	39.4%	17.1%	37.8%	$ 1,835	$ 56	$ 3,264	18.3%
1999	5.5%	40.6%	16.2%	37.6%	$ 1,611	$ 48	$ 3,076	17.6%
1998	5.4%	40.4%	16.0%	38.2%	$ 1,414	$ 40	$ 2,651	15.5%
1997	5.2%	41.2%	15.7%	37.9%	$ 1,306	$ 36	$ 2,566	14.4%
1996	5.5%	42.9%	16.5%	35.1%	$ 1,226	$ 32	$ 2,202	13.1%

© 2021 Holle M et al. *JAMA Network Open.*

HHE								
Year	Top 50%	Next 49%	Next 1%	Non-spenders	Cutoff_50	Cutoff_49	Mean_50	%_total
2018	0.7%	9.5%	5.7%	84.1%	$ 17,281	$ 332	$ 36,549	8.8%
2017	0.5%	4.4%	3.1%	92.1%	$ 19,382	$ 573	$ 41,600	7.9%
2016	0.3%	4.4%	3.3%	92.0%	$ 21,177	$ 335	$ 43,938	6.9%
2015	0.3%	4.2%	3.3%	92.2%	$ 22,559	$ 345	$ 43,482	6.4%
2014	0.3%	4.0%	3.2%	92.6%	$ 18,180	$ 299	$ 36,059	6.0%
2013	0.4%	4.3%	3.0%	92.3%	$ 14,427	$ 226	$ 28,962	5.0%
2012	0.3%	3.6%	2.6%	93.6%	$ 21,757	$ 300	$ 40,373	5.4%
2011	0.3%	3.1%	2.2%	94.3%	$ 16,178	$ 409	$ 30,646	5.1%
2010	0.3%	3.1%	2.3%	94.2%	$ 11,627	$ 287	$ 24,842	4.2%
2009	0.3%	3.1%	2.3%	94.2%	$ 19,324	$ 328	$ 37,311	5.3%
2008	0.3%	3.2%	2.7%	93.7%	$ 16,976	$ 436	$ 29,335	5.5%
2007	0.3%	3.2%	2.4%	94.1%	$ 14,017	$ 232	$ 28,331	4.7%
2006	0.4%	3.2%	2.4%	94.0%	$ 10,042	$ 215	$ 19,019	4.5%
2005	0.2%	3.3%	2.9%	93.6%	$ 16,321	$ 275	$ 28,966	5.8%
2004	0.2%	3.2%	2.4%	94.2%	$ 14,173	$ 369	$ 24,689	5.0%
2003	0.3%	3.5%	2.6%	93.6%	$ 10,180	$ 160	$ 20,067	4.6%
2002	0.3%	3.1%	2.4%	94.2%	$ 13,572	$ 250	$ 28,577	5.9%
2001	0.2%	3.2%	2.1%	94.4%	$ 14,998	$ 240	$ 32,397	5.8%
2000	0.2%	2.8%	2.0%	95.0%	$ 15,807	$ 250	$ 29,622	5.3%
1999	0.1%	2.7%	2.2%	95.0%	$ 28,992	$ 365	$ 49,262	7.4%
1998	0.2%	3.2%	2.3%	94.3%	$ 11,240	$ 130	$ 26,944	5.4%
1997	0.3%	3.3%	2.8%	93.6%	$ 13,025	$ 231	$ 22,816	7.7%
1996	0.3%	3.3%	3.0%	93.4%	$ 15,273	$ 249	$ 26,317	8.4%

© 2021 Holle M et al. *JAMA Network Open.*

Any Private							
Year	Top 50%	Next 49%	Next 1%	Non-spenders	Cutoff_50	Cutoff_49	Mean_50
2018	3.9%	55.8%	22.5%	17.9%	$ 24,190	$ 418	$ 59,503
2017	3.7%	54.7%	21.9%	19.7%	$ 20,610	$ 350	$ 51,221
2016	3.9%	54.4%	21.2%	20.4%	$ 18,473	$ 340	$ 45,303
2015	3.7%	54.9%	22.4%	19.0%	$ 20,063	$ 334	$ 46,811
2014	3.7%	55.6%	22.1%	18.6%	$ 17,066	$ 326	$ 44,126
2013	4.2%	55.4%	22.1%	18.3%	$ 17,229	$ 318	$ 41,865
2012	3.7%	55.1%	22.6%	18.6%	$ 18,667	$ 328	$ 45,941
2011	4.4%	55.3%	22.2%	18.2%	$ 16,511	$ 314	$ 38,420
2010	4.3%	56.0%	21.6%	18.1%	$ 14,626	$ 301	$ 36,050
2009	4.4%	55.8%	21.9%	17.8%	$ 14,283	$ 308	$ 35,083
2008	4.7%	55.6%	20.8%	18.9%	$ 12,198	$ 289	$ 29,160
2007	4.1%	55.8%	21.5%	18.6%	$ 13,721	$ 278	$ 33,703
2006	4.5%	55.9%	20.9%	18.7%	$ 11,549	$ 254	$ 28,025
2005	4.5%	56.5%	19.8%	19.1%	$ 10,907	$ 253	$ 27,467
2004	4.7%	55.6%	20.4%	19.3%	$ 9,963	$ 238	$ 23,982
2003	4.5%	57.2%	20.0%	18.3%	$ 9,188	$ 220	$ 23,317
2002	4.6%	56.8%	20.1%	18.5%	$ 8,271	$ 199	$ 20,679
2001	4.8%	56.9%	19.1%	19.2%	$ 7,491	$ 182	$ 17,935
2000	3.8%	55.3%	19.1%	21.8%	$ 8,079	$ 160	$ 19,340

© 2021 Holle M et al. *JAMA Network Open.*

**Any Medicare**							
**Year**	**Top 50%**	**Next 49%**	**Next 1%**	**Non-spenders**	**Cutoff_50**	**Cutoff_49**	**Mean_50**
2018	9.9%	68.7%	17.8%	3.6%	$ 32,144	$ 1,419	$ 64,487
2017	9.0%	67.7%	19.4%	3.8%	$ 33,113	$ 1,285	$ 67,326
2016	9.5%	68.5%	18.4%	3.7%	$ 31,688	$ 1,264	$ 60,419
2015	9.1%	68.6%	18.8%	3.6%	$ 31,514	$ 1,246	$ 62,259
2014	9.8%	67.7%	18.8%	3.7%	$ 28,909	$ 1,302	$ 61,116
2013	10.3%	68.3%	17.2%	4.2%	$ 24,091	$ 1,170	$ 49,200
2012	9.9%	67.9%	18.7%	3.5%	$ 24,122	$ 1,026	$ 48,056
2011	9.4%	68.8%	17.9%	3.9%	$ 25,389	$ 1,058	$ 49,806
2010	9.4%	68.8%	18.0%	3.8%	$ 26,024	$ 1,186	$ 53,157
2009	9.5%	68.0%	18.3%	4.2%	$ 25,705	$ 1,127	$ 52,839
2008	10.9%	68.3%	16.6%	4.2%	$ 22,602	$ 1,103	$ 43,455
2007	10.1%	68.6%	16.7%	4.6%	$ 22,177	$ 1,142	$ 45,645
2006	10.1%	70.3%	16.0%	3.6%	$ 21,187	$ 1,092	$ 42,978
2005	9.0%	70.5%	16.6%	3.9%	$ 23,751	$ 1,092	$ 45,976
2004	9.2%	70.3%	17.0%	3.6%	$ 22,815	$ 1,086	$ 45,024
2003	9.0%	69.2%	17.5%	4.4%	$ 21,549	$ 926	$ 42,427
2002	9.6%	69.2%	16.5%	4.8%	$ 18,769	$ 912	$ 38,347
2001	8.9%	67.8%	18.3%	5.0%	$ 17,929	$ 763	$ 37,160
2000	7.9%	67.6%	18.6%	5.9%	$ 18,252	$ 651	$ 34,562

© 2021 Holle M et al. *JAMA Network Open.*

FFS							
**Year**	**Top 50%**	**Next 49%**	**Next 1%**	**Non-spenders**	**Cutoff_50**	**Cutoff_49**	**Mean_50**
2018	10.1%	69.5%	17.1%	3.4%	$ 29,323	$ 1,357	$ 58,442
2017	9.1%	68.1%	18.9%	3.8%	$ 29,513	$ 1,190	$ 61,417
2016	9.4%	69.7%	17.6%	3.3%	$ 30,554	$ 1,236	$ 58,626
2015	8.6%	69.2%	18.9%	3.3%	$ 31,394	$ 1,214	$ 62,638
2014	10.2%	67.3%	18.5%	4.0%	$ 26,341	$ 1,303	$ 52,493
2013	10.5%	67.6%	16.7%	5.2%	$ 22,513	$ 1,131	$ 46,627
2012	10.3%	67.9%	18.2%	3.6%	$ 24,083	$ 1,077	$ 46,067
2011	9.7%	69.2%	17.2%	3.8%	$ 24,816	$ 1,058	$ 49,813
2010	9.8%	69.6%	16.8%	3.7%	$ 23,370	$ 1,212	$ 51,668
2009	9.7%	69.1%	17.1%	4.0%	$ 23,260	$ 1,098	$ 47,227
2008	10.7%	69.3%	15.9%	4.2%	$ 20,592	$ 1,090	$ 42,148
2007	10.5%	69.5%	16.0%	4.0%	$ 22,184	$ 1,195	$ 45,179
2006	10.2%	71.0%	15.4%	3.4%	$ 21,516	$ 1,126	$ 43,358

© 2021 Holle M et al. *JAMA Network Open.*

MA							
**Year**	**Top 50%**	**Next 49%**	**Next 1%**	**Non-spenders**	**Cutoff_50**	**Cutoff_49**	**Mean_50**
2018	9.9%	70.2%	17.4%	2.5%	$ 29,369	$ 1,319	$ 61,339
2017	9.2%	69.9%	17.8%	3.1%	$ 30,254	$ 1,234	$ 59,165
2016	9.1%	69.9%	18.1%	2.9%	$ 29,887	$ 1,210	$ 58,564
2015	9.7%	70.0%	17.1%	3.3%	$ 27,129	$ 1,132	$ 54,052
2014	9.3%	70.7%	17.9%	2.1%	$ 27,818	$ 1,179	$ 58,985
2013	9.8%	70.6%	16.6%	2.9%	$ 23,187	$ 1,064	$ 46,064
2012	10.6%	69.4%	17.7%	2.3%	$ 20,022	$ 905	$ 40,368
2011	9.3%	70.5%	17.6%	2.6%	$ 21,720	$ 980	$ 43,792
2010	8.7%	69.5%	18.5%	3.3%	$ 27,461	$ 1,139	$ 55,043
2009	8.9%	69.2%	18.3%	3.6%	$ 26,328	$ 1,051	$ 55,208
2008	11.3%	70.2%	15.2%	3.2%	$ 20,521	$ 1,082	$ 40,087
2007	9.6%	70.6%	15.6%	4.2%	$ 19,580	$ 961	$ 41,905
2006	10.6%	72.3%	14.1%	3.1%	$ 18,631	$ 1,018	$ 36,706

© 2021 Holle M et al. *JAMA Network Open.*

MCAID							
**Year**	**Top 50%**	**Next 49%**	**Next 1%**	**Non-spenders**	**Cutoff_50**	**Cutoff_49**	**Mean_50**
2018	4.1%	48.8%	28.2%	18.8%	$ 34,109	$ 483	$ 74,227
2017	3.8%	46.6%	28.7%	20.9%	$ 34,874	$ 491	$ 71,656
2016	3.4%	45.5%	29.5%	21.6%	$ 34,065	$ 433	$ 69,096
2015	3.6%	48.2%	28.6%	19.6%	$ 30,043	$ 379	$ 65,349
2014	3.5%	48.2%	29.8%	18.5%	$ 31,771	$ 378	$ 69,453
2013	4.0%	48.9%	28.8%	18.4%	$ 26,393	$ 387	$ 54,711
2012	3.4%	49.1%	30.0%	17.5%	$ 26,016	$ 322	$ 62,063
2011	3.4%	48.1%	29.7%	18.8%	$ 25,725	$ 322	$ 63,779
2010	3.4%	49.0%	29.0%	18.6%	$ 26,235	$ 304	$ 59,704
2009	3.5%	48.7%	29.8%	18.1%	$ 30,393	$ 334	$ 61,671
2008	3.9%	46.6%	30.4%	19.1%	$ 26,149	$ 340	$ 55,920
2007	3.4%	46.3%	29.5%	20.8%	$ 24,746	$ 306	$ 54,853
2006	4.5%	47.9%	27.3%	20.3%	$ 17,821	$ 291	$ 39,532
2005	3.4%	47.6%	29.6%	19.3%	$ 24,763	$ 324	$ 51,108
2004	3.4%	49.6%	28.3%	18.7%	$ 20,612	$ 281	$ 44,261
2003	4.2%	51.8%	26.8%	17.3%	$ 17,847	$ 263	$ 38,418
2002	3.7%	49.7%	27.6%	19.0%	$ 20,657	$ 283	$ 47,704
2001	4.1%	49.9%	27.4%	18.6%	$ 15,047	$ 226	$ 32,531
2000	3.1%	46.3%	30.6%	19.9%	$ 22,932	$ 251	$ 45,900

© 2021 Holle M et al. *JAMA Network Open.*

Dual							
Year	Top 50%	Next 49%	Next 1%	Non-spenders	Cutoff_50	Cutoff_49	Mean_50
2018	11.8%	63.5%	20.2%	4.5%	$ 47,696	$ 2,347	$ 85,752
2017	11.6%	62.7%	22.0%	3.7%	$ 45,129	$ 2,261	$ 84,891
2016	11.5%	63.4%	20.2%	5.0%	$ 43,197	$ 2,212	$ 74,900
2015	10.8%	63.5%	22.3%	3.3%	$ 39,478	$ 1,632	$ 74,957
2014	10.8%	64.5%	20.3%	4.3%	$ 45,110	$ 2,050	$ 90,480
2013	11.7%	66.7%	19.2%	2.4%	$ 35,915	$ 1,849	$ 64,003
2012	9.2%	64.7%	22.7%	3.4%	$ 35,806	$ 1,525	$ 73,194
2011	11.7%	62.5%	20.2%	5.7%	$ 30,866	$ 1,682	$ 52,241
2010	10.5%	64.1%	21.0%	4.4%	$ 33,532	$ 1,593	$ 61,328
2009	11.0%	59.7%	23.0%	6.2%	$ 37,801	$ 2,102	$ 70,992
2008	13.1%	59.0%	22.0%	5.9%	$ 33,365	$ 1,652	$ 50,333
2007	9.8%	62.0%	21.6%	6.6%	$ 29,812	$ 1,511	$ 60,725
2006	10.6%	63.7%	20.2%	5.4%	$ 23,472	$ 1,296	$ 46,723
2005	8.2%	65.6%	20.6%	5.5%	$ 37,091	$ 1,594	$ 63,225
2004	7.8%	65.2%	22.3%	4.7%	$ 32,963	$ 1,340	$ 60,855
2003	11.1%	61.7%	20.1%	7.1%	$ 19,390	$ 1,071	$ 36,363
2002	10.6%	60.4%	20.1%	9.0%	$ 25,928	$ 1,253	$ 48,450
2001	10.2%	61.2%	21.7%	7.0%	$ 21,428	$ 1,090	$ 39,091
2000	7.8%	60.0%	26.4%	5.8%	$ 30,883	$ 1,107	$ 57,114

© 2021 Holle M et al. *JAMA Network Open.*

Uninsured							
**Year**	**Top 50%**	**Next 49%**	**Next 1%**	**Non-spenders**	**Cutoff_50**	**Cutoff_49**	**Mean_50**
2018	1.6%	32.5%	12.6%	53.3%	$ 12,845	$ 160	$ 32,170
2017	1.6%	30.6%	13.7%	54.1%	$ 12,375	$ 177	$ 45,352
2016	1.5%	28.4%	16.3%	53.7%	$ 22,557	$ 248	$ 48,684
2015	2.0%	30.7%	14.6%	52.8%	$ 12,113	$ 168	$ 31,248
2014	0.6%	29.7%	16.6%	53.1%	$ 28,302	$ 200	$ 83,106
2013	1.9%	32.3%	15.6%	50.3%	$ 13,345	$ 179	$ 32,360
2012	2.7%	31.9%	15.3%	50.1%	$ 9,358	$ 163	$ 20,941
2011	1.8%	33.1%	15.3%	49.8%	$ 11,439	$ 150	$ 31,387
2010	1.8%	30.6%	16.1%	51.5%	$ 13,083	$ 181	$ 29,782
2009	2.1%	32.6%	15.4%	49.9%	$ 9,386	$ 154	$ 26,741
2008	2.9%	33.0%	14.0%	50.1%	$ 7,126	$ 142	$ 16,680
2007	2.3%	34.0%	15.2%	48.5%	$ 9,243	$ 148	$ 24,395
2006	3.0%	33.6%	13.0%	50.4%	$ 6,036	$ 127	$ 14,386
2005	1.8%	35.0%	14.0%	49.1%	$ 8,260	$ 142	$ 25,842
2004	2.2%	33.8%	13.1%	50.9%	$ 6,521	$ 112	$ 15,971
2003	2.6%	34.7%	13.5%	49.2%	$ 6,203	$ 115	$ 15,594
2002	2.4%	35.2%	13.8%	48.6%	$ 5,159	$ 110	$ 16,268
2001	1.6%	36.5%	15.3%	46.6%	$ 7,872	$ 117	$ 22,890
2000	1.5%	33.6%	15.3%	49.6%	$ 8,268	$ 113	$ 23,574

© 2021 Holle M et al. *JAMA Network Open.*

**Diabetics**							
**Year**	**Top 50%**	**Next 49%**	**Next 1%**	**Non-spenders**	**Cutoff_50**	**Cutoff_49**	**Mean_50**
2018	10.6%	67.9%	18.6%	2.9%	$ 38,243	$ 1,927	$ 78,351
2017	10.1%	68.0%	19.6%	2.3%	$ 33,878	$ 1,615	$ 70,694
2016	10.6%	68.4%	18.7%	2.4%	$ 32,788	$ 1,528	$ 62,129
2015	9.9%	68.7%	18.9%	2.6%	$ 33,977	$ 1,639	$ 69,371
2014	9.6%	69.2%	19.3%	1.9%	$ 33,113	$ 1,569	$ 71,821
2013	9.2%	69.7%	18.8%	2.2%	$ 29,373	$ 1,448	$ 62,839
2012	10.3%	69.1%	18.6%	2.0%	$ 26,872	$ 1,306	$ 57,178
2011	11.1%	68.9%	18.3%	1.8%	$ 25,278	$ 1,195	$ 49,994
2010	10.0%	70.2%	17.7%	2.0%	$ 27,879	$ 1,386	$ 55,382
2009	10.5%	69.7%	17.7%	2.2%	$ 26,498	$ 1,351	$ 52,745
2008	10.5%	70.5%	16.5%	2.5%	$ 24,433	$ 1,275	$ 49,889
2007	9.7%	72.1%	16.1%	2.1%	$ 26,078	$ 1,292	$ 55,630
2006	11.3%	72.6%	14.9%	1.2%	$ 23,024	$ 1,276	$ 46,073
2005	9.8%	73.7%	15.2%	1.3%	$ 25,536	$ 1,289	$ 50,656
2004	10.3%	74.1%	13.8%	1.9%	$ 21,202	$ 1,254	$ 44,954
2003	11.9%	72.8%	13.6%	1.6%	$ 18,793	$ 1,227	$ 37,106
2002	11.3%	72.8%	14.9%	1.0%	$ 18,409	$ 1,087	$ 38,256
2001	9.6%	73.2%	15.6%	1.6%	$ 20,781	$ 976	$ 40,136
2000	10.2%	72.8%	15.1%	1.8%	$ 16,372	$ 779	$ 31,239

© 2021 Holle M et al. *JAMA Network Open.*

IncGrp1							
Year	Top 50%	Next 49%	Next 1%	Non-spenders	Cutoff_50	Cutoff_49	Mean_50
2018	4.4%	45.7%	26.9%	23.0%	$ 32,753	$ 569	$ 70,785
2017	4.5%	44.9%	25.8%	24.8%	$ 29,429	$ 494	$ 59,784
2016	4.1%	43.2%	27.5%	25.2%	$ 30,670	$ 454	$ 64,318
2015	4.3%	45.3%	25.3%	25.1%	$ 24,720	$ 401	$ 52,711
2014	3.8%	44.9%	27.1%	24.2%	$ 28,120	$ 419	$ 57,957
2013	4.1%	45.3%	25.3%	25.4%	$ 23,099	$ 368	$ 50,543
2012	3.8%	45.4%	26.0%	24.7%	$ 20,953	$ 320	$ 49,937
2011	3.4%	45.3%	26.4%	24.8%	$ 23,056	$ 351	$ 58,421
2010	3.3%	43.6%	26.2%	26.9%	$ 25,174	$ 358	$ 58,266
2009	3.3%	44.1%	25.4%	27.2%	$ 23,140	$ 321	$ 49,798
2008	3.5%	43.5%	25.2%	27.8%	$ 22,122	$ 354	$ 50,382
2007	3.4%	44.1%	26.5%	26.0%	$ 24,045	$ 352	$ 52,255
2006	4.1%	44.9%	25.3%	25.7%	$ 17,794	$ 320	$ 38,855
2005	4.2%	44.0%	24.9%	27.0%	$ 18,471	$ 317	$ 39,286
2004	3.1%	44.7%	25.5%	26.6%	$ 21,365	$ 317	$ 47,733
2003	4.6%	45.6%	23.6%	26.2%	$ 13,987	$ 284	$ 30,690
2002	4.1%	45.8%	23.9%	26.1%	$ 15,880	$ 290	$ 36,309
2001	3.6%	45.4%	24.5%	26.5%	$ 15,495	$ 241	$ 32,732
2000	3.1%	43.4%	25.3%	28.2%	$ 16,331	$ 226	$ 33,445

© 2021 Holle M et al. *JAMA Network Open.*

IncGrp2							
**Year**	**Top 50%**	**Next 49%**	**Next 1%**	**Non-spenders**	**Cutoff_50**	**Cutoff_49**	**Mean_50**
2018	4.7%	49.2%	24.4%	21.7%	$ 33,580	$ 526	$ 63,793
2017	5.1%	45.4%	26.4%	23.1%	$ 31,826	$ 591	$ 60,942
2016	4.4%	46.0%	26.0%	23.6%	$ 25,760	$ 465	$ 47,594
2015	4.0%	48.0%	25.7%	22.3%	$ 27,509	$ 434	$ 65,281
2014	4.1%	46.2%	28.0%	21.7%	$ 38,183	$ 596	$ 77,137
2013	4.6%	45.0%	26.0%	24.3%	$ 23,479	$ 408	$ 47,253
2012	3.7%	45.6%	27.0%	23.7%	$ 33,470	$ 516	$ 68,091
2011	3.9%	44.7%	25.4%	26.1%	$ 22,825	$ 407	$ 53,544
2010	4.4%	44.7%	24.4%	26.5%	$ 21,942	$ 385	$ 44,302
2009	5.1%	44.4%	26.2%	24.3%	$ 25,612	$ 461	$ 45,130
2008	4.8%	45.3%	24.6%	25.3%	$ 22,448	$ 417	$ 42,266
2007	4.1%	44.9%	24.4%	26.6%	$ 24,746	$ 369	$ 47,309
2006	4.6%	46.4%	25.8%	23.1%	$ 23,834	$ 431	$ 47,750
2005	3.9%	45.2%	24.1%	26.7%	$ 22,520	$ 393	$ 53,239
2004	4.3%	44.7%	23.5%	27.5%	$ 17,758	$ 316	$ 38,950
2003	4.2%	47.0%	23.2%	25.6%	$ 20,604	$ 337	$ 40,793
2002	3.7%	49.1%	22.9%	24.3%	$ 16,750	$ 274	$ 38,863
2001	4.3%	46.8%	23.6%	25.3%	$ 16,161	$ 291	$ 33,897
2000	4.3%	47.3%	21.2%	27.1%	$ 12,398	$ 250	$ 26,232

© 2021 Holle M et al. *JAMA Network Open.*

IncGrp3							
**Year**	**Top 50%**	**Next 49%**	**Next 1%**	**Non-spenders**	**Cutoff_50**	**Cutoff_49**	**Mean_50**
2018	4.4%	48.6%	25.8%	21.2%	$ 29,834	$ 491	$ 65,299
2017	4.2%	46.6%	24.2%	25.1%	$ 28,362	$ 521	$ 60,713
2016	3.8%	45.7%	24.7%	25.8%	$ 29,555	$ 492	$ 60,493
2015	3.5%	46.0%	27.5%	23.0%	$ 32,440	$ 482	$ 68,326
2014	3.5%	45.6%	25.3%	25.6%	$ 28,553	$ 419	$ 64,447
2013	4.6%	45.4%	23.5%	26.4%	$ 21,454	$ 395	$ 43,482
2012	3.7%	46.9%	24.9%	24.4%	$ 22,291	$ 381	$ 54,396
2011	4.2%	47.2%	24.0%	24.6%	$ 19,996	$ 364	$ 44,647
2010	3.8%	47.2%	24.6%	24.5%	$ 21,281	$ 347	$ 50,479
2009	3.9%	45.0%	24.7%	26.5%	$ 24,013	$ 421	$ 52,859
2008	4.5%	45.5%	24.0%	26.0%	$ 19,582	$ 374	$ 42,147
2007	3.9%	43.8%	24.9%	27.5%	$ 22,411	$ 399	$ 50,671
2006	4.2%	45.0%	22.1%	28.7%	$ 17,039	$ 331	$ 36,799
2005	3.4%	47.8%	24.1%	24.8%	$ 22,369	$ 338	$ 48,558
2004	4.4%	46.6%	23.9%	25.0%	$ 17,721	$ 313	$ 35,518
2003	4.2%	48.3%	22.5%	25.1%	$ 15,347	$ 309	$ 34,756
2002	3.9%	47.6%	23.5%	25.1%	$ 16,022	$ 295	$ 37,524
2001	4.0%	47.7%	22.9%	25.4%	$ 14,354	$ 263	$ 32,379
2000	3.4%	46.5%	23.8%	26.3%	$ 14,545	$ 231	$ 34,920

© 2021 Holle M et al. *JAMA Network Open.*

IncGrp4							
**Year**	**Top 50%**	**Next 49%**	**Next 1%**	**Non-spenders**	**Cutoff_50**	**Cutoff_49**	**Mean_50**
2018	4.2%	51.7%	24.0%	20.2%	$ 27,539	$ 491	$ 62,056
2017	3.9%	51.4%	23.9%	20.8%	$ 23,742	$ 406	$ 57,171
2016	3.7%	50.1%	23.2%	23.0%	$ 24,649	$ 404	$ 56,714
2015	3.8%	50.0%	23.6%	22.6%	$ 24,288	$ 397	$ 52,398
2014	4.2%	50.9%	23.5%	21.4%	$ 18,617	$ 350	$ 44,593
2013	3.8%	50.6%	23.9%	21.7%	$ 22,707	$ 412	$ 53,723
2012	4.2%	50.8%	23.3%	21.8%	$ 19,722	$ 366	$ 44,552
2011	4.5%	51.0%	23.1%	21.5%	$ 18,748	$ 357	$ 39,475
2010	4.4%	50.2%	23.1%	22.3%	$ 18,169	$ 352	$ 40,849
2009	3.9%	50.4%	23.9%	21.8%	$ 19,710	$ 363	$ 44,961
2008	4.5%	50.9%	21.4%	23.2%	$ 15,766	$ 327	$ 35,089
2007	4.4%	50.2%	22.4%	23.1%	$ 16,021	$ 307	$ 35,461
2006	4.8%	50.6%	21.6%	23.0%	$ 13,469	$ 280	$ 29,378
2005	3.9%	51.2%	22.0%	22.8%	$ 16,110	$ 302	$ 35,563
2004	4.4%	50.7%	21.7%	23.2%	$ 13,111	$ 276	$ 29,337
2003	3.6%	52.9%	22.3%	21.2%	$ 15,007	$ 274	$ 36,892
2002	4.0%	52.5%	22.0%	21.4%	$ 12,224	$ 240	$ 29,983
2001	4.6%	52.6%	21.6%	21.3%	$ 10,396	$ 222	$ 24,598
2000	3.9%	51.6%	20.8%	23.8%	$ 10,547	$ 200	$ 24,027

© 2021 Holle M et al. *JAMA Network Open.*

IncGrp5							
**Year**	**Top 50%**	**Next 49%**	**Next 1%**	**Non-spenders**	**Cutoff_50**	**Cutoff_49**	**Mean_50**
2018	5.2%	58.1%	22.2%	14.5%	$ 24,355	$ 550	$ 55,875
2017	4.5%	56.3%	23.0%	16.2%	$ 24,155	$ 473	$ 55,856
2016	5.1%	57.1%	21.7%	16.1%	$ 20,176	$ 460	$ 46,559
2015	4.8%	57.2%	23.1%	15.0%	$ 22,587	$ 450	$ 50,472
2014	4.9%	57.8%	22.3%	14.9%	$ 19,273	$ 456	$ 45,431
2013	5.4%	57.3%	22.0%	15.3%	$ 17,483	$ 407	$ 38,733
2012	5.1%	56.7%	22.4%	15.7%	$ 18,260	$ 417	$ 41,834
2011	5.0%	56.9%	22.8%	15.3%	$ 20,102	$ 424	$ 44,978
2010	5.0%	57.7%	22.2%	15.1%	$ 17,222	$ 393	$ 39,785
2009	5.6%	58.6%	21.5%	14.4%	$ 14,960	$ 386	$ 35,123
2008	5.8%	57.3%	21.7%	15.2%	$ 13,680	$ 376	$ 30,505
2007	5.0%	57.6%	21.9%	15.4%	$ 14,039	$ 339	$ 34,197
2006	5.0%	58.3%	21.2%	15.5%	$ 13,729	$ 338	$ 34,351
2005	5.2%	58.8%	20.2%	15.8%	$ 12,256	$ 325	$ 30,216
2004	5.0%	57.8%	21.4%	15.9%	$ 12,542	$ 309	$ 29,926
2003	5.0%	58.8%	20.8%	15.4%	$ 10,875	$ 276	$ 25,869
2002	5.4%	58.2%	20.7%	15.8%	$ 9,538	$ 253	$ 23,269
2001	4.8%	59.4%	19.9%	15.9%	$ 8,795	$ 225	$ 22,313
2000	4.0%	56.7%	19.9%	19.4%	$ 9,491	$ 195	$ 22,862

© 2021 Holle M et al. *JAMA Network Open.*

eAppendix 2: Prescription Drug Expenditure Concentration Parameters for Population Subgroups	
**Introduction**	
These tables present data on prescription drug expenditure concentration parameters for different population groups between 1996-2018. These tables are also available as Microsoft Excel files from a public repository (https://github.com/recon-strategy/concentration-parameters).	
The "Summaries" sheet provides an overview of the studied population subgroups.	
**Contents**	
Variable descriptions	
Summaries	
2018 Comparison	
US_pop	
US_pop_0_65	
US_pop_65+	
Any_private	
Any_Medicare	
FFS	
MA	
MCAID	
Dual	
Unins	
Diabetics	
IncGrp1	
IncGrp2	
IncGrp3	
IncGrp4	
IncGrp5	

© 2021 Holle M et al. *JAMA Network Open.*

Variable Descriptions

Variable	Description
Top 50%	Percentage of the population whose cumulative expenditures make up the top 50th percentile of expenditure
Next 49%	Percentage of the population whose cumulative expenditures make up the 49th-1st expenditure percentiles
Next 1%	Percentage of the population whose cumulative expenditures make up the 1st expenditure percentile
Non-spenders	Percentage of the population with no expenditures
Cutoff_50[a]	Lowest individual expenditure in the top 50% of cumulative expenditure
Cutoff_49[a]	Lowest individual expenditure in the top 99% of cumulative expenditure
Mean_50[a]	Mean expenditure of the top 50% cumulative expenditure bracket
%_total	Expenditure category share of total expenditure (where applicable)

[a]*not adjusted for inflation*

© 2021 Holle M et al. *JAMA Network Open.*

Summaries		
**Subgroup/expenditure category**	**Population**	**Expenditure variable**
US_pop	Full US population	Prescription drug expenditure
US_Pop (0-64)	US Population (0-64)	Prescription drug expenditure
US_Pop (65+)	US Population (65+)	Prescription drug expenditure
any_private	Individual had any private insurance coverage at any time during the year and was not covered by public insurance	Prescription drug expenditure
any_medicare	Covered by Medicare	Prescription drug expenditure
FFS	Covered by Medicare but not Medicare Managed Care or Medicaid	Prescription drug expenditure
MA	Covered by Medicare Managed Care/Medicare Advantage	Prescription drug expenditure

© 2021 Holle M et al. *JAMA Network Open.*

MCAID	Covered by Medicaid or SCHIP	Prescription drug expenditure
Dual	Covered by both Medicare and Medicaid at time of interview	Prescription drug expenditure
Unins	Individual was uninsured during all of 2018	Prescription drug expenditure
Diabetics	Diabetes diagnosis at time of interview (pre 2018 only over 18s asked)	Prescription drug expenditure
IncGrp1	Family income less than 100% of poverty line	Prescription drug expenditure
IncGrp2	Family income between 100% - 125% of poverty line	Prescription drug expenditure
IncGrp3	Family income between 125% - 199% of poverty line	Prescription drug expenditure
IncGrp4	Family income between 200% - 399% of poverty line	Prescription drug expenditure
IncGrp5	Family income greater than 400% of poverty line	Prescription drug expenditure
Note: for some subgroups, data starts after 1996 due to methodological changes/data availability.		

© 2021 Holle M et al. *JAMA Network Open.*

**2018 Comparison**								
**Segment**	**Year**	**Top 50%**	**Next 49%**	**Next 1%**	**Non-spenders**	**Cutoff_50**	**Cutoff_49**	**Mean_50**
US_Pop	2018	2.3%	33.8%	24.2%	39.8%	$ 13,182	$ 175	$ 30,523
US_Pop (0-65)	2018	1.5%	29.0%	23.7%	45.8%	$ 14,714	$ 139	$ 34,564
US_Pop (65+)	2018	6.2%	61.4%	22.7%	9.7%	$ 11,104	$ 302	$ 24,786
any_private	2018	1.4%	31.5%	23.4%	43.7%	$ 13,182	$ 124	$ 34,857
any_medicare	2018	6.3%	61.5%	23.6%	8.7%	$ 12,523	$ 348	$ 28,905
FFS	2018	6.0%	62.7%	23.0%	8.4%	$ 11,276	$ 305	$ 24,900
MA	2018	6.5%	62.4%	23.5%	7.6%	$ 13,418	$ 406	$ 29,809
MCAID	2018	2.5%	28.2%	26.2%	43.1%	$ 15,779	$ 229	$ 32,780
Dual	2018	8.6%	57.2%	25.3%	8.9%	$ 22,451	$ 671	$ 40,332
Unins	2018	0.4%	16.7%	11.8%	71.1%	$ 12,845	$ 50	$ 30,462
Diabetics	2018	11.5%	60.5%	22.7%	5.2%	$ 13,418	$ 626	$ 25,350
IncGrp1	2018	2.6%	30.2%	25.2%	41.9%	$ 17,590	$ 248	$ 34,726
IncGrp2	2018	2.1%	30.0%	24.9%	43.0%	$ 16,562	$ 212	$ 40,392
IncGrp3	2018	2.6%	32.6%	23.1%	41.6%	$ 12,409	$ 184	$ 26,769
IncGrp4	2018	2.0%	31.7%	23.2%	43.1%	$ 13,515	$ 173	$ 31,606
IncGrp5	2018	2.3%	37.1%	24.5%	36.1%	$ 11,911	$ 164	$ 28,619

© 2021 Holle M et al. *JAMA Network Open.*

US_pop							
Year	Top 50%	Next 49%	Next 1%	Non-spenders	Cutoff_50	Cutoff_49	Mean_50
2018	2.3%	33.8%	24.2%	39.8%	$ 13,182	$ 175	$ 30,523
2017	2.2%	33.9%	23.7%	40.2%	$ 11,973	$ 165	$ 29,136
2016	2.7%	34.3%	23.2%	39.7%	$ 9,530	$ 163	$ 21,573
2015	2.8%	34.8%	23.7%	38.7%	$ 8,745	$ 152	$ 19,628
2014	2.8%	34.8%	23.8%	38.6%	$ 8,198	$ 140	$ 18,375
2013	3.2%	34.6%	22.9%	39.3%	$ 6,914	$ 127	$ 15,395
2012	3.2%	35.5%	23.7%	37.6%	$ 6,335	$ 122	$ 15,325
2011	3.3%	35.9%	23.4%	37.3%	$ 6,151	$ 128	$ 15,988
2010	4.2%	35.0%	22.0%	38.7%	$ 4,902	$ 125	$ 10,988
2009	4.4%	36.6%	21.6%	37.5%	$ 4,771	$ 112	$ 9,852
2008	5.0%	36.5%	20.8%	37.7%	$ 4,215	$ 118	$ 8,142
2007	5.0%	37.0%	19.8%	38.3%	$ 3,943	$ 112	$ 7,785
2006	5.2%	39.3%	18.1%	37.4%	$ 3,459	$ 105	$ 6,783
2005	5.6%	40.0%	17.5%	36.9%	$ 3,155	$ 102	$ 6,184
2004	6.2%	39.4%	17.1%	37.3%	$ 2,809	$ 90	$ 5,416
2003	6.1%	41.1%	17.2%	35.6%	$ 2,740	$ 81	$ 5,164
2002	6.2%	41.0%	17.2%	35.6%	$ 2,315	$ 75	$ 4,436
2001	6.0%	41.6%	17.1%	35.3%	$ 2,115	$ 68	$ 3,897
2000	5.7%	39.4%	17.1%	37.8%	$ 1,835	$ 56	$ 3,264
1999	5.5%	40.6%	16.2%	37.6%	$ 1,611	$ 48	$ 3,076
1998	5.4%	40.4%	16.0%	38.2%	$ 1,414	$ 40	$ 2,651
1997	5.2%	41.2%	15.7%	37.9%	$ 1,306	$ 36	$ 2,566
1996	5.5%	42.9%	16.5%	35.1%	$ 1,226	$ 32	$ 2,202

© 2021 Holle M et al. *JAMA Network Open.*

US_pop							
**Year**	**Top 50%**	**Next 49%**	**Next 1%**	**Non-spenders**	**Cutoff_50**	**Cutoff_49**	**Mean_50**
2018	1.5%	29.0%	23.7%	45.8%	$ 14,714	$ 139	$ 34,564
2017	1.4%	29.4%	23.1%	46.0%	$ 14,046	$ 132	$ 32,776
2016	2.0%	30.2%	22.4%	45.4%	$ 9,530	$ 121	$ 21,734
2015	1.9%	30.8%	23.3%	43.9%	$ 10,240	$ 123	$ 22,996
2014	1.7%	30.8%	23.6%	43.8%	$ 9,603	$ 110	$ 22,358
2013	2.0%	31.0%	22.6%	44.4%	$ 8,193	$ 99	$ 18,836
2012	2.1%	31.9%	23.6%	42.4%	$ 7,107	$ 97	$ 19,180
2011	2.1%	32.6%	23.3%	42.0%	$ 6,948	$ 102	$ 20,984
2010	3.0%	32.4%	21.5%	43.2%	$ 4,821	$ 92	$ 12,129
2009	3.2%	34.1%	20.9%	41.9%	$ 4,735	$ 87	$ 10,500
2008	3.7%	34.2%	20.2%	41.9%	$ 4,147	$ 88	$ 8,431
2007	3.7%	34.9%	19.0%	42.4%	$ 3,651	$ 84	$ 7,859
2006	3.8%	37.4%	17.3%	41.5%	$ 3,330	$ 80	$ 7,176
2005	4.3%	38.2%	16.7%	40.9%	$ 3,071	$ 79	$ 6,270
2004	4.8%	37.8%	16.0%	41.4%	$ 2,611	$ 72	$ 5,339
2003	4.7%	39.8%	16.0%	39.4%	$ 2,496	$ 64	$ 5,101
2002	4.8%	39.5%	16.3%	39.3%	$ 2,060	$ 59	$ 4,415
2001	4.8%	40.2%	16.2%	38.9%	$ 1,884	$ 54	$ 3,750
2000	4.5%	37.8%	16.3%	41.4%	$ 1,667	$ 44	$ 3,171
1999	4.3%	39.5%	14.9%	41.2%	$ 1,400	$ 36	$ 2,944
1998	4.3%	39.2%	14.7%	41.8%	$ 1,257	$ 30	$ 2,534
1997	4.1%	40.2%	14.5%	41.2%	$ 1,201	$ 29	$ 2,526
1996	4.7%	42.0%	15.1%	38.3%	$ 954	$ 25	$ 1,935

© 2021 Holle M et al. *JAMA Network Open.*

US_pop (0-65)							
Year	Top 50%	Next 49%	Next 1%	Non-spenders	Cutoff_50	Cutoff_49	Mean_50
2018	6.2%	61.4%	22.7%	9.7%	$ 11,104	$ 302	$ 24,786
2017	6.3%	61.1%	23.0%	9.6%	$ 9,624	$ 310	$ 22,886
2016	7.0%	61.7%	21.9%	9.3%	$ 9,093	$ 341	$ 21,161
2015	8.6%	60.9%	21.3%	9.2%	$ 7,364	$ 314	$ 15,179
2014	9.7%	62.1%	19.3%	8.9%	$ 6,332	$ 276	$ 12,275
2013	10.4%	61.5%	18.6%	9.5%	$ 6,005	$ 302	$ 11,214
2012	10.3%	61.2%	19.5%	9.0%	$ 5,244	$ 241	$ 9,941
2011	11.3%	61.3%	19.0%	8.3%	$ 5,349	$ 274	$ 9,307
2010	12.6%	60.6%	17.5%	9.2%	$ 5,189	$ 327	$ 9,317
2009	12.6%	61.2%	17.9%	8.3%	$ 4,788	$ 294	$ 8,615
2008	14.5%	62.0%	15.2%	8.3%	$ 4,253	$ 329	$ 7,304
2007	14.3%	62.3%	14.6%	8.8%	$ 4,402	$ 345	$ 7,543
2006	16.0%	63.4%	13.1%	7.5%	$ 3,614	$ 355	$ 6,024
2005	15.2%	63.8%	13.1%	7.9%	$ 3,638	$ 331	$ 5,986
2004	16.8%	62.7%	13.4%	7.1%	$ 3,259	$ 308	$ 5,438
2003	16.5%	62.3%	13.3%	7.9%	$ 3,312	$ 300	$ 5,181
2002	16.8%	62.2%	12.9%	8.1%	$ 2,505	$ 248	$ 4,339
2001	16.0%	60.6%	13.8%	9.6%	$ 2,472	$ 217	$ 4,034
2000	15.1%	60.1%	14.2%	10.6%	$ 2,057	$ 174	$ 3,393
1999	15.4%	60.1%	14.0%	10.5%	$ 1,996	$ 186	$ 3,141
1998	14.6%	60.1%	14.1%	11.2%	$ 1,654	$ 125	$ 2,719
1997	13.9%	58.9%	14.0%	13.3%	$ 1,628	$ 118	$ 2,629
1996	13.5%	59.5%	15.0%	12.0%	$ 1,579	$ 109	$ 2,610

© 2021 Holle M et al. *JAMA Network Open.*

Any Private							
**Year**	**Top 50%**	**Next 49%**	**Next 1%**	**Non-spenders**	**Cutoff_50**	**Cutoff_49**	**Mean_50**
2018	1.4%	31.5%	23.4%	43.7%	$ 13,182	$ 124	$ 34,857
2017	1.4%	32.0%	23.1%	43.6%	$ 11,284	$ 119	$ 32,250
2016	2.0%	33.1%	22.2%	42.8%	$ 8,075	$ 110	$ 19,747
2015	2.1%	34.0%	22.9%	41.0%	$ 7,787	$ 105	$ 17,615
2014	1.5%	34.5%	23.7%	40.3%	$ 9,554	$ 105	$ 23,939
2013	2.1%	34.6%	22.9%	40.5%	$ 6,714	$ 94	$ 17,006
2012	2.4%	35.3%	23.6%	38.6%	$ 6,062	$ 91	$ 14,241
2011	2.9%	37.1%	22.8%	37.2%	$ 4,963	$ 86	$ 12,315
2010	4.0%	36.3%	21.2%	38.5%	$ 3,536	$ 88	$ 7,814
2009	3.9%	38.0%	20.8%	37.3%	$ 4,010	$ 86	$ 8,586
2008	4.5%	38.2%	19.7%	37.6%	$ 3,223	$ 85	$ 6,577
2007	4.5%	39.1%	18.8%	37.6%	$ 3,155	$ 81	$ 6,410
2006	4.7%	41.3%	16.7%	37.3%	$ 2,658	$ 76	$ 5,644
2005	5.2%	42.0%	16.0%	36.8%	$ 2,431	$ 75	$ 5,141
2004	6.2%	41.3%	15.4%	37.2%	$ 2,167	$ 70	$ 3,905
2003	6.0%	43.0%	15.4%	35.6%	$ 2,146	$ 68	$ 3,780
2002	5.7%	43.0%	16.1%	35.2%	$ 1,722	$ 57	$ 3,426
2001	5.9%	43.1%	15.6%	35.5%	$ 1,576	$ 53	$ 2,894
2000	5.4%	40.5%	15.7%	38.4%	$ 1,309	$ 42	$ 2,435

© 2021 Holle M et al. *JAMA Network Open.*

Any Medicare							
Year	Top 50%	Next 49%	Next 1%	Non-spenders	Cutoff_50	Cutoff_49	Mean_50
2018	6.3%	61.5%	23.6%	8.7%	$ 12,523	$ 348	$ 28,905
2017	5.9%	60.8%	24.4%	8.9%	$ 13,246	$ 353	$ 28,440
2016	6.9%	62.4%	22.6%	8.1%	$ 11,793	$ 383	$ 24,510
2015	7.1%	62.0%	22.6%	8.2%	$ 9,707	$ 357	$ 21,846
2014	8.8%	62.7%	20.4%	8.2%	$ 8,227	$ 338	$ 17,414
2013	9.7%	62.8%	19.4%	8.1%	$ 7,312	$ 314	$ 14,465
2012	9.6%	61.5%	20.8%	8.0%	$ 6,450	$ 267	$ 13,267
2011	10.0%	61.6%	20.5%	8.0%	$ 6,874	$ 293	$ 13,384
2010	11.5%	61.4%	18.4%	8.7%	$ 6,219	$ 347	$ 11,847
2009	11.4%	61.6%	18.6%	8.4%	$ 5,688	$ 347	$ 11,048
2008	13.1%	61.6%	16.0%	9.3%	$ 5,149	$ 362	$ 8,995
2007	13.2%	61.7%	15.3%	9.8%	$ 4,800	$ 393	$ 8,718
2006	13.6%	64.3%	14.3%	7.8%	$ 4,241	$ 422	$ 7,751
2005	14.3%	63.8%	13.7%	8.2%	$ 4,181	$ 373	$ 7,138
2004	15.3%	62.6%	14.2%	8.0%	$ 3,840	$ 327	$ 6,611
2003	14.6%	62.5%	14.2%	8.7%	$ 3,552	$ 300	$ 6,409
2002	15.4%	61.6%	13.4%	9.6%	$ 2,996	$ 279	$ 5,012
2001	14.6%	60.4%	14.7%	10.3%	$ 3,036	$ 208	$ 4,785
2000	14.5%	59.7%	14.8%	11.0%	$ 2,447	$ 185	$ 3,838

© 2021 Holle M et al. *JAMA Network Open.*

FFS							
**Year**	**Top 50%**	**Next 49%**	**Next 1%**	**Non-spenders**	**Cutoff_50**	**Cutoff_49**	**Mean_50**
2018	6.0%	62.7%	23.0%	8.4%	$ 11,276	$ 305	$ 24,900
2017	6.8%	61.3%	22.6%	9.3%	$ 9,758	$ 275	$ 21,623
2016	6.6%	62.6%	23.3%	7.5%	$ 10,876	$ 383	$ 24,199
2015	6.4%	62.6%	22.7%	8.4%	$ 9,707	$ 369	$ 22,999
2014	10.2%	62.2%	19.1%	8.5%	$ 7,197	$ 315	$ 13,421
2013	10.2%	62.4%	17.9%	9.6%	$ 6,707	$ 304	$ 13,178
2012	10.8%	61.7%	19.0%	8.6%	$ 5,514	$ 291	$ 11,080
2011	10.1%	62.1%	20.2%	7.6%	$ 6,896	$ 350	$ 14,524
2010	12.6%	62.2%	17.3%	7.9%	$ 6,277	$ 370	$ 11,033
2009	11.8%	62.3%	17.6%	8.2%	$ 5,266	$ 302	$ 9,803
2008	12.7%	62.2%	15.7%	9.4%	$ 5,149	$ 362	$ 9,166
2007	13.3%	62.7%	15.0%	9.0%	$ 4,631	$ 429	$ 8,764
2006	13.2%	65.1%	14.3%	7.5%	$ 4,679	$ 422	$ 7,855

© 2021 Holle M et al. *JAMA Network Open.*

MA							
**Year**	**Top 50%**	**Next 49%**	**Next 1%**	**Non-spenders**	**Cutoff_50**	**Cutoff_49**	**Mean_50**
2018	6.5%	62.4%	23.5%	7.6%	$ 13,418	$ 406	$ 29,809
2017	5.6%	61.4%	25.6%	7.4%	$ 14,764	$ 365	$ 30,527
2016	6.9%	63.7%	21.8%	7.6%	$ 11,260	$ 403	$ 25,033
2015	7.9%	63.2%	21.6%	7.3%	$ 9,990	$ 399	$ 19,722
2014	7.0%	64.8%	22.0%	6.3%	$ 10,048	$ 338	$ 21,296
2013	8.8%	64.3%	21.1%	5.8%	$ 7,612	$ 331	$ 14,149
2012	9.0%	63.6%	21.6%	5.8%	$ 7,114	$ 267	$ 12,949
2011	11.0%	62.5%	19.9%	6.6%	$ 5,862	$ 283	$ 10,174
2010	12.0%	60.7%	19.3%	8.0%	$ 5,381	$ 298	$ 9,802
2009	11.0%	62.3%	19.2%	7.4%	$ 6,501	$ 372	$ 11,115
2008	14.5%	63.3%	15.7%	6.5%	$ 4,374	$ 408	$ 7,587
2007	14.4%	62.9%	14.5%	8.1%	$ 4,273	$ 299	$ 6,955
2006	16.2%	64.8%	12.9%	6.1%	$ 3,633	$ 355	$ 6,100

© 2021 Holle M et al. *JAMA Network Open.*

MCAID							
**Year**	**Top 50%**	**Next 49%**	**Next 1%**	**Non-spenders**	**Cutoff_50**	**Cutoff_49**	**Mean_50**
2018	2.5%	28.2%	26.2%	43.1%	$ 15,779	$ 229	$ 32,780
2017	2.3%	28.7%	25.8%	43.1%	$ 15,705	$ 242	$ 33,508
2016	2.9%	27.9%	25.5%	43.8%	$ 13,264	$ 210	$ 25,133
2015	2.9%	29.0%	26.3%	41.8%	$ 10,901	$ 170	$ 22,234
2014	2.5%	27.5%	27.9%	42.0%	$ 12,986	$ 211	$ 26,250
2013	3.2%	29.6%	26.3%	40.9%	$ 9,165	$ 151	$ 18,683
2012	2.6%	29.3%	28.9%	39.2%	$ 10,084	$ 151	$ 28,383
2011	1.7%	28.1%	28.5%	41.7%	$ 13,649	$ 195	$ 50,161
2010	2.5%	28.0%	27.0%	42.4%	$ 9,568	$ 159	$ 23,874
2009	3.3%	30.7%	26.3%	39.7%	$ 6,956	$ 129	$ 14,686
2008	4.0%	28.5%	27.5%	39.9%	$ 7,051	$ 128	$ 12,267
2007	4.2%	29.1%	24.7%	42.0%	$ 6,016	$ 136	$ 10,589
2006	4.7%	32.1%	24.6%	38.6%	$ 4,988	$ 105	$ 8,979
2005	4.9%	33.0%	23.9%	38.2%	$ 5,195	$ 109	$ 8,880
2004	4.3%	34.0%	23.3%	38.5%	$ 5,071	$ 104	$ 9,139
2003	4.4%	37.3%	24.0%	34.2%	$ 4,740	$ 83	$ 8,578
2002	4.9%	35.2%	22.7%	37.3%	$ 3,810	$ 81	$ 7,451
2001	4.7%	36.3%	23.0%	36.0%	$ 3,784	$ 68	$ 6,259
2000	4.7%	36.2%	23.8%	35.3%	$ 3,140	$ 64	$ 5,457

© 2021 Holle M et al. *JAMA Network Open.*

Dual							
**Year**	**Top 50%**	**Next 49%**	**Next 1%**	**Non-spenders**	**Cutoff_50**	**Cutoff_49**	**Mean_50**
2018	8.6%	57.2%	25.3%	8.9%	$ 22,451	$ 671	$ 40,332
2017	7.3%	58.7%	25.7%	8.3%	$ 20,379	$ 804	$ 40,724
2016	9.9%	59.7%	21.3%	9.1%	$ 15,838	$ 711	$ 30,009
2015	9.6%	60.2%	23.1%	7.1%	$ 14,221	$ 609	$ 25,682
2014	9.5%	61.0%	22.2%	7.4%	$ 16,919	$ 762	$ 29,237
2013	11.6%	63.1%	18.7%	6.7%	$ 10,573	$ 624	$ 19,705
2012	9.5%	59.3%	23.3%	7.9%	$ 11,435	$ 576	$ 24,330
2011	10.1%	57.2%	21.3%	11.4%	$ 11,473	$ 556	$ 18,972
2010	9.7%	60.5%	18.7%	11.1%	$ 10,795	$ 668	$ 19,293
2009	12.0%	57.1%	19.6%	11.3%	$ 8,456	$ 766	$ 15,963
2008	14.8%	54.5%	16.4%	14.4%	$ 7,944	$ 617	$ 11,630
2007	14.2%	54.5%	17.2%	14.1%	$ 6,437	$ 670	$ 10,226
2006	14.3%	56.2%	17.4%	12.1%	$ 5,586	$ 504	$ 8,969
2005	14.3%	58.6%	16.3%	10.9%	$ 6,133	$ 579	$ 9,756
2004	12.2%	59.0%	18.9%	10.0%	$ 7,165	$ 481	$ 11,278
2003	12.7%	55.3%	18.9%	13.1%	$ 6,343	$ 462	$ 9,888
2002	15.1%	54.9%	14.6%	15.4%	$ 4,705	$ 468	$ 7,017
2001	13.8%	54.4%	18.5%	13.3%	$ 4,744	$ 360	$ 6,914
2000	13.6%	55.4%	20.2%	10.8%	$ 4,818	$ 337	$ 6,062

© 2021 Holle M et al. *JAMA Network Open.*

Uninsured							
**Year**	**Top 50%**	**Next 49%**	**Next 1%**	**Non-spenders**	**Cutoff_50**	**Cutoff_49**	**Mean_50**
2018	0.4%	16.7%	11.8%	71.1%	$ 12,845	$ 50	$ 30,462
2017	1.1%	18.1%	10.9%	69.9%	$ 4,708	$ 55	$ 10,626
2016	1.4%	17.3%	13.9%	67.4%	$ 6,607	$ 71	$ 13,374
2015	0.9%	18.9%	12.9%	67.3%	$ 7,316	$ 62	$ 17,232
2014	1.3%	20.2%	11.3%	67.2%	$ 4,754	$ 42	$ 8,692
2013	0.9%	21.6%	13.7%	63.8%	$ 6,354	$ 52	$ 13,437
2012	1.5%	22.5%	13.4%	62.6%	$ 4,269	$ 45	$ 9,149
2011	1.6%	21.8%	13.0%	63.7%	$ 3,857	$ 43	$ 6,981
2010	1.8%	21.2%	12.0%	65.0%	$ 3,391	$ 46	$ 6,101
2009	1.8%	23.1%	12.3%	62.8%	$ 2,340	$ 37	$ 5,162
2008	2.1%	22.6%	12.5%	62.7%	$ 3,349	$ 57	$ 6,521
2007	2.3%	22.8%	11.5%	63.5%	$ 2,429	$ 44	$ 5,016
2006	2.9%	23.6%	10.2%	63.2%	$ 2,030	$ 55	$ 4,320
2005	2.7%	24.9%	9.6%	62.8%	$ 2,003	$ 52	$ 3,842
2004	2.6%	23.1%	9.4%	64.8%	$ 1,967	$ 41	$ 3,534
2003	2.7%	24.8%	9.1%	63.4%	$ 1,768	$ 40	$ 3,434
2002	2.8%	25.1%	10.2%	61.9%	$ 1,770	$ 40	$ 3,177
2001	2.9%	26.5%	11.3%	59.4%	$ 1,390	$ 33	$ 2,630
2000	2.9%	23.5%	11.2%	62.5%	$ 1,420	$ 36	$ 2,531

© 2021 Holle M et al. *JAMA Network Open.*

**Diabetics**							
**Year**	**Top 50%**	**Next 49%**	**Next 1%**	**Non-spenders**	**Cutoff_50**	**Cutoff_49**	**Mean_50**
2018	11.5%	60.5%	22.7%	5.2%	$ 13,418	$ 626	$ 25,350
2017	9.8%	62.5%	22.9%	4.7%	$ 13,949	$ 547	$ 26,741
2016	9.9%	64.8%	20.7%	4.6%	$ 13,793	$ 609	$ 26,644
2015	9.2%	65.3%	20.9%	4.5%	$ 13,523	$ 567	$ 25,266
2014	10.5%	64.3%	20.7%	4.4%	$ 10,834	$ 508	$ 20,401
2013	11.7%	64.7%	19.3%	4.3%	$ 8,787	$ 495	$ 16,393
2012	12.6%	63.4%	20.2%	3.9%	$ 7,363	$ 400	$ 13,101
2011	12.6%	64.8%	19.1%	3.4%	$ 6,896	$ 410	$ 13,075
2010	15.4%	63.6%	17.0%	4.0%	$ 6,219	$ 488	$ 10,872
2009	14.0%	65.5%	16.9%	3.6%	$ 6,536	$ 433	$ 11,584
2008	15.1%	65.7%	14.6%	4.6%	$ 6,107	$ 481	$ 10,244
2007	16.1%	67.2%	13.7%	2.9%	$ 5,693	$ 554	$ 9,441
2006	17.6%	67.9%	11.9%	2.6%	$ 5,606	$ 498	$ 9,000
2005	18.1%	67.6%	11.3%	3.0%	$ 4,876	$ 524	$ 7,771
2004	19.3%	66.4%	11.6%	2.8%	$ 4,763	$ 513	$ 7,381
2003	19.0%	67.1%	11.4%	2.6%	$ 4,411	$ 548	$ 7,240
2002	19.5%	66.5%	12.0%	1.9%	$ 4,037	$ 399	$ 5,931
2001	17.7%	66.1%	13.2%	3.1%	$ 3,833	$ 346	$ 5,918
2000	18.1%	67.0%	11.6%	3.3%	$ 3,069	$ 278	$ 4,694

© 2021 Holle M et al. *JAMA Network Open.*

IncGrp1							
**Year**	**Top 50%**	**Next 49%**	**Next 1%**	**Non-spenders**	**Cutoff_50**	**Cutoff_49**	**Mean_50**
2018	2.6%	30.2%	25.2%	41.9%	$ 17,590	$ 248	$ 34,726
2017	2.1%	29.8%	23.6%	44.4%	$ 17,018	$ 208	$ 34,400
2016	2.7%	28.6%	24.5%	44.2%	$ 12,593	$ 211	$ 25,412
2015	2.7%	29.0%	25.6%	42.8%	$ 10,901	$ 180	$ 23,387
2014	2.9%	29.0%	24.7%	43.4%	$ 10,559	$ 165	$ 20,847
2013	2.6%	30.1%	23.3%	44.0%	$ 8,302	$ 127	$ 18,938
2012	2.3%	29.9%	25.3%	42.6%	$ 8,123	$ 123	$ 24,083
2011	1.4%	29.1%	26.4%	43.0%	$ 11,473	$ 168	$ 48,535
2010	3.3%	28.3%	22.9%	45.5%	$ 6,036	$ 115	$ 12,093
2009	3.2%	30.0%	21.9%	44.8%	$ 5,475	$ 110	$ 11,100
2008	4.1%	30.4%	22.5%	43.0%	$ 5,515	$ 104	$ 9,384
2007	4.2%	30.9%	21.3%	43.5%	$ 5,247	$ 114	$ 9,321
2006	4.8%	32.2%	21.0%	42.0%	$ 4,535	$ 105	$ 7,641
2005	4.6%	32.5%	20.0%	42.9%	$ 4,562	$ 107	$ 7,774
2004	4.4%	33.0%	19.6%	43.0%	$ 4,369	$ 100	$ 7,926
2003	4.8%	35.2%	19.4%	40.6%	$ 3,885	$ 88	$ 6,770
2002	5.2%	34.5%	18.1%	42.2%	$ 3,383	$ 79	$ 6,236
2001	4.6%	35.6%	19.4%	40.3%	$ 3,281	$ 70	$ 5,281
2000	4.5%	33.7%	20.3%	41.5%	$ 2,636	$ 54	$ 4,472

© 2021 Holle M et al. *JAMA Network Open.*

IncGrp2							
Year	Top 50%	Next 49%	Next 1%	Non-spenders	Cutoff_50	Cutoff_49	Mean_50
2018	2.1%	30.0%	24.9%	43.0%	$ 16,562	$ 212	$ 40,392
2017	2.1%	31.9%	26.6%	39.3%	$ 20,901	$ 271	$ 44,044
2016	3.2%	32.3%	23.5%	41.0%	$ 11,730	$ 228	$ 23,578
2015	3.4%	33.4%	23.4%	39.8%	$ 9,787	$ 178	$ 17,387
2014	4.4%	35.3%	23.5%	36.8%	$ 7,758	$ 160	$ 14,102
2013	3.4%	31.2%	25.1%	40.3%	$ 9,912	$ 156	$ 17,657
2012	2.3%	33.5%	25.6%	38.5%	$ 12,131	$ 190	$ 31,455
2011	3.3%	30.6%	22.9%	43.1%	$ 6,896	$ 163	$ 17,459
2010	3.9%	30.5%	22.3%	43.3%	$ 6,915	$ 184	$ 12,627
2009	4.0%	32.1%	24.0%	39.9%	$ 7,266	$ 169	$ 14,127
2008	5.2%	32.9%	22.1%	39.8%	$ 5,375	$ 142	$ 8,688
2007	5.4%	33.5%	18.8%	42.3%	$ 4,321	$ 141	$ 8,272
2006	5.5%	37.1%	21.3%	36.1%	$ 4,842	$ 145	$ 8,501
2005	5.6%	34.5%	18.5%	41.4%	$ 4,318	$ 132	$ 6,843
2004	5.3%	33.4%	19.8%	41.5%	$ 4,277	$ 116	$ 7,581
2003	4.9%	38.0%	19.4%	37.7%	$ 4,120	$ 131	$ 7,846
2002	6.1%	36.9%	19.1%	37.8%	$ 3,337	$ 93	$ 5,084
2001	5.9%	37.2%	17.8%	39.1%	$ 2,622	$ 75	$ 4,247
2000	5.8%	36.8%	17.5%	39.9%	$ 2,656	$ 70	$ 3,691

© 2021 Holle M et al. *JAMA Network Open.*

IncGrp3							
Year	Top 50%	Next 49%	Next 1%	Non-spenders	Cutoff_50	Cutoff_49	Mean_50
2018	2.6%	32.6%	23.1%	41.6%	$ 12,409	$ 184	$ 26,769
2017	2.6%	31.7%	22.3%	43.4%	$ 10,845	$ 192	$ 25,297
2016	3.2%	31.5%	22.0%	43.2%	$ 10,253	$ 198	$ 20,553
2015	2.5%	31.7%	23.6%	42.1%	$ 10,604	$ 214	$ 24,000
2014	2.7%	30.4%	23.2%	43.8%	$ 9,410	$ 162	$ 20,489
2013	3.4%	31.1%	21.6%	43.9%	$ 7,612	$ 132	$ 14,592
2012	3.6%	32.8%	22.4%	41.2%	$ 5,644	$ 108	$ 11,892
2011	3.3%	33.1%	21.8%	41.8%	$ 5,906	$ 130	$ 13,224
2010	3.5%	32.2%	22.4%	42.0%	$ 6,991	$ 146	$ 14,734
2009	3.8%	33.2%	21.5%	41.5%	$ 5,932	$ 111	$ 11,055
2008	4.3%	33.1%	20.8%	41.7%	$ 5,196	$ 128	$ 9,906
2007	5.0%	32.8%	19.3%	42.9%	$ 4,631	$ 132	$ 8,051
2006	5.7%	33.8%	17.4%	43.1%	$ 3,217	$ 103	$ 6,145
2005	4.4%	37.2%	18.3%	40.2%	$ 4,187	$ 109	$ 7,850
2004	5.8%	36.2%	17.5%	40.5%	$ 3,831	$ 105	$ 6,335
2003	5.5%	37.9%	17.5%	39.0%	$ 3,310	$ 89	$ 5,926
2002	5.5%	36.9%	17.8%	39.8%	$ 2,767	$ 84	$ 5,080
2001	5.8%	36.9%	17.4%	39.8%	$ 2,678	$ 76	$ 4,446
2000	5.4%	36.2%	18.0%	40.4%	$ 2,167	$ 64	$ 3,733

© 2021 Holle M et al. *JAMA Network Open.*

IncGrp4							
**Year**	**Top 50%**	**Next 49%**	**Next 1%**	**Non-spenders**	**Cutoff_50**	**Cutoff_49**	**Mean_50**
2018	2.0%	31.7%	23.2%	43.1%	$ 13,515	$ 173	$ 31,606
2017	2.7%	33.3%	22.9%	41.2%	$ 8,777	$ 144	$ 19,127
2016	2.6%	33.0%	22.4%	42.1%	$ 8,370	$ 147	$ 19,241
2015	2.4%	33.4%	23.3%	40.9%	$ 9,803	$ 143	$ 21,075
2014	2.7%	33.5%	23.1%	40.8%	$ 7,062	$ 118	$ 16,369
2013	2.9%	33.7%	22.8%	40.6%	$ 7,164	$ 120	$ 14,786
2012	2.7%	35.0%	23.7%	38.7%	$ 7,023	$ 115	$ 14,097
2011	3.7%	35.2%	22.9%	38.3%	$ 5,082	$ 111	$ 11,203
2010	3.9%	34.0%	22.2%	39.8%	$ 5,380	$ 117	$ 12,536
2009	3.8%	35.6%	21.5%	39.1%	$ 5,089	$ 108	$ 11,022
2008	4.9%	35.4%	20.2%	39.6%	$ 3,862	$ 107	$ 7,336
2007	4.5%	35.7%	19.4%	40.5%	$ 4,047	$ 107	$ 8,120
2006	5.0%	38.3%	17.1%	39.6%	$ 3,207	$ 94	$ 6,506
2005	5.5%	39.1%	17.2%	38.3%	$ 3,183	$ 92	$ 6,014
2004	6.1%	38.3%	16.6%	39.0%	$ 2,764	$ 85	$ 5,002
2003	6.0%	40.0%	16.9%	37.1%	$ 2,532	$ 77	$ 4,720
2002	5.6%	40.6%	17.0%	36.8%	$ 2,286	$ 66	$ 4,275
2001	6.1%	41.0%	16.8%	36.0%	$ 2,115	$ 65	$ 3,787
2000	5.6%	39.0%	17.3%	38.1%	$ 1,888	$ 54	$ 3,248

© 2021 Holle M et al. *JAMA Network Open.*

IncGrp5							
**Year**	**Top 50%**	**Next 49%**	**Next 1%**	**Non-spenders**	**Cutoff_50**	**Cutoff_49**	**Mean_50**
2018	2.3%	37.1%	24.5%	36.1%	$ 11,911	$ 164	$ 28,619
2017	1.8%	36.7%	24.1%	37.3%	$ 12,080	$ 165	$ 35,839
2016	2.8%	38.2%	23.6%	35.4%	$ 9,141	$ 160	$ 21,431
2015	3.4%	39.0%	23.1%	34.4%	$ 7,294	$ 140	$ 16,059
2014	2.8%	39.7%	24.0%	33.5%	$ 7,900	$ 149	$ 19,067
2013	3.5%	39.0%	23.0%	34.5%	$ 6,265	$ 138	$ 14,488
2012	4.3%	39.5%	23.1%	33.1%	$ 5,768	$ 130	$ 11,582
2011	4.4%	41.0%	23.1%	31.5%	$ 5,322	$ 125	$ 12,027
2010	5.5%	40.0%	21.3%	33.1%	$ 4,061	$ 123	$ 8,043
2009	5.8%	41.7%	21.1%	31.5%	$ 4,089	$ 117	$ 7,744
2008	5.7%	41.5%	20.3%	32.4%	$ 4,064	$ 130	$ 7,668
2007	5.7%	41.8%	19.8%	32.7%	$ 3,444	$ 112	$ 6,827
2006	5.6%	44.5%	17.8%	32.1%	$ 3,309	$ 110	$ 6,784
2005	7.0%	44.6%	16.5%	31.9%	$ 2,672	$ 104	$ 5,092
2004	7.8%	43.9%	16.2%	32.1%	$ 2,199	$ 90	$ 4,032
2003	7.3%	45.3%	16.1%	31.3%	$ 2,411	$ 88	$ 4,213
2002	7.6%	45.1%	16.6%	30.7%	$ 1,922	$ 76	$ 3,474
2001	6.9%	45.6%	16.3%	31.2%	$ 1,694	$ 70	$ 3,153
2000	6.6%	42.6%	15.6%	35.2%	$ 1,464	$ 54	$ 2,617

© 2021 Holle M et al. *JAMA Network Open.*

eAppendix 3: Expenditure Category Shares of Total Expenditure 1996-2018	
**Introduction**	
The data presented show trends in expenditure category shares of total expenditure between 1996 and 2018.<br>This table is also available as Microsoft Excel files from a public repository (https://github.com/recon-strategy/concentration-parameters).	
**Contents**	
Expenditure category shares	

**Expenditure category shares**					
**Year**	**OP**	**IP**	**ED**	**RX**	**HHE**
2018	37.9%	24.7%	3.7%	24.8%	8.8%
2017	36.1%	26.4%	4.0%	25.6%	7.9%
2016	35.9%	27.7%	4.2%	25.4%	6.9%
2015	35.3%	28.6%	4.6%	25.1%	6.4%
2014	36.8%	27.4%	4.7%	25.1%	6.0%
2013	36.4%	30.2%	4.6%	23.8%	5.0%
2012	34.1%	32.6%	4.5%	23.4%	5.4%
2011	35.2%	31.4%	4.2%	24.1%	5.1%
2010	34.9%	33.5%	4.1%	23.2%	4.2%
2009	36.4%	31.7%	4.4%	22.1%	5.3%
2008	35.1%	31.4%	4.5%	23.5%	5.5%
2007	34.8%	34.2%	3.7%	22.5%	4.7%
2006	35.4%	32.5%	4.0%	23.6%	4.5%
2005	34.3%	33.7%	3.5%	22.7%	5.8%
2004	34.7%	34.4%	4.2%	21.7%	5.0%
2003	32.8%	37.3%	3.6%	21.7%	4.6%
2002	35.1%	34.8%	3.8%	20.4%	5.9%
2001	34.2%	35.7%	3.9%	20.4%	5.8%
Note: total expenditure does not include vision aids or dental health care					

© 2021 Holle M et al. *JAMA Network Open.*
